# Analysis of MADS-Box Gene Family Reveals Conservation in Floral Organ ABCDE Model of Moso Bamboo (*Phyllostachys edulis*)

**DOI:** 10.3389/fpls.2017.00656

**Published:** 2017-05-03

**Authors:** Zhanchao Cheng, Wei Ge, Long Li, Dan Hou, Yanjun Ma, Jun Liu, Qingsong Bai, Xueping Li, Shaohua Mu, Jian Gao

**Affiliations:** Key Laboratory of Bamboo and Rattan Science and Technology of the State Forestry Administration, International Centre for Bamboo and RattanBeijing, China

**Keywords:** *Phyllostachys edulis*, MADS-box, floral organ, ABCDE model, *PheMADS15*

## Abstract

Mini chromosome maintenance 1, agamous, deficiens, and serum response factor (MADS)-box genes are transcription factors which play fundamental roles in flower development and regulation of floral organ identity. However, till date, identification and functions of MADS-box genes remain largely unclear in *Phyllostachys edulis*. In view of this, we performed a whole-genome survey and identified 34 MADS-box genes in *P. edulis*, and based on phylogeny, they were classified as MIKC^C^, MIKC^∗^, Mα, and Mβ. The detailed analysis about gene structure and motifs, phylogenetic classification, comparison of gene divergence and duplication are provided. Interestingly, expression patterns for most genes were found similar to those of *Arabidopsis* and rice, indicating that the well-established ABCDE model can be applied to *P. edulis*. Moreover, we overexpressed *PheMADS15*, an *AP1*-like gene, in *Arabidopsis*, and found that the transgenic plants have early flowering phenotype, suggesting that *PheMADS15* might be a regulator of flowering transition in *P. edulis*. Taken together, this study provides not only insightful comprehension but also useful information for understanding the functions of MADS-box genes in *P. edulis*.

## Introduction

*Phyllostachys edulis* is one of the most important non-timber forest products in the world. They flower at the end of very long vegetative growth phases, often followed by the death of large areas of *P. edulis*. They show a cyclic recurrence of flowering, the intervals of which are basically definite varying from a few years to 120 years or longer. In this case, studying the mechanism of *P. edulis* flowering time is very challenging, and it is quite difficult to determine the key regulatory genes involved in floral formation and transition in *P. edulis*.

The mini chromosome maintenance 1, agamous, deficiens, and serum response factor (MADS)-box family members, identified originally as floral homeotic genes, are important transcription factors for plant development (Purugganan, 1997; Theissen et al., 2000; Jack, 2001; Nam et al., 2003; Pařenicová et al., 2003). In plants, the type I comprises M-type genes and type II group includes most well-known MIKC-type genes (Arora et al., 2007), named after the four basic components of the MADS (M) domain: the Intervening (I) domain, the Keratin (K) domain, and the C-terminal (C) domain (Kramer et al., 1998). MIKC-type genes have been further divided into two subgroups, MIKC^C^- and MIKC^∗^-types, due to different exon/intron structures (Henschel et al., 2002; Kofuji et al., 2003). Type I MADS-box genes have been categorized into Mα, Mβ, Mγ, and Mδ clades based on the phylogenetic relationships of conserved MADS-box domain (Pařenicová et al., 2003). In most plants, type I genes experience a higher number of births and deaths than type II genes, due to more frequent segmental gene duplications and weaker purifying selection (Nam et al., 2004).

The genetic ABCDE model of floral organ development can be applied to dicot plants, mainly in *Arabidopsis*, snapdragon and petunia (Coen and Meyerowitz, 1991; Angenent and Colombo, 1996; Theißen and Saedler, 2001). Generally, A and B class genes together are required for petal development, B and C class genes cooperate to control stamen development. A and C class genes are, respectively, involved in sepal and carpel development. D class genes function in ovule development, while E class proteins are expressed in all four whorls of floral organs by forming MADS-box protein complexes with proteins of other classes (Pelaz et al., 2000; Favaro et al., 2003; Pinyopich et al., 2003). In *Arabidopsis*, *APETALA1* (*AP1*) and *APETALA2* (*AP2*) belong to the A-function genes; B-function genes include *APETALA3* (*AP3*), *PISTILLATA* (*PI*); the C-function gene is *AGAMOUS* (*AG*); *SEPALLATA1*, *2*, *3* and *4* (*SEP1*, *2*, *3*, *4*/*AGL2*, *4*, *9*, *3*) form the E-function genes (Yanofsky et al., 1990; Jack et al., 1992; Mandel et al., 1992; Goto and Meyerowitz, 1994; Jofuku et al., 1994; Huang et al., 1995; Savidge et al., 1995; Mandel and Yanofsky, 1998; Pelaz et al., 2000; Ditta et al., 2004).

Poaceae family is generally known for monocot crops such as rice (*Oryza sativa*), maize (*Zea mays*), wheat (*Triticum* spp.) and barley (*Hordeum vulgare*) (Grass Phylogeny Working Group et al., 2001). However, Bambusoideae is quite distinct from other members of Poaceae and is known for its unique floral organization and morphology (Grass Phylogeny Working Group et al., 2001; Rudall et al., 2005; Whipple et al., 2007). In rice and bamboo, each grass spikelet is the structural unit of grass flowers, which consists of a number of florets. In addition, the floret contains four whorls, such as lemma and palea (whorl 1), two lodicules (whorl 2), six stamens (whorl 3), and gynoecium (whorl 4) (Nagasawa et al., 2003). Like eudicots, MADS-box genes in rice and maize are divided into ABCDE gene classes. (Ambrose et al., 2000; Nagasawa et al., 2003; Kater et al., 2006; Yamaguchi et al., 2006; Preston and Kellogg, 2007; Yao et al., 2008; Cui et al., 2010; Li et al., 2011). Few of the MADS-box genes are functionally characterized in different plants. For instance, three *AP1*/*FUL*-like genes (*OsMADS14*, *OsMADS15*, and *OsMADS18*) coordinately act in the shoot apical meristem in rice (Kobayashi et al., 2012). Maize *Silky1* and rice *SPW1* or Os*MADS16*, orthologs of the *Arabidopsis AP3*, cause homeotic transformations of stamens to carpels and lodicules to lemma- or palea-like structures (Ambrose et al., 2000; Nagasawa et al., 2003; Whipple et al., 2004). Rice *OsMADS3*, belonging to the *AG* homolog gene, is, respectively, involved in stamen, ovule and late anther development (Kramer et al., 2004; Yamaguchi et al., 2006; Hu et al., 2011; Li et al., 2011). In rice, *OsMADS13* and *OsMADS21*, two D class genes, are involved in ovule identity specification and floral meristem termination (Dreni et al., 2007; Li et al., 2011). Simultaneous four rice *SEP*-like genes, *LHS1*, *OsMADS5*, *OsMADS7*, and *OsMADS8*, play a key role in all floral organs development (Cui et al., 2010). However, for bamboo, especially *P. edulis*, fewer genes were reported to play roles in specifying flower development, their genetic interactions remained to be answered (Tian et al., 2005, 2006; Li et al., 2009; Shih et al., 2014). Therefore, it is necessary to systematically study MADS-box genes related to flower development in bamboo.

In *Arabidopsis*, class A genes are represented by *AP1* (Mandel et al., 1992) and *AP2* (Jofuku et al., 1994). *AP1* and *LEAFY* (*LFY*) are floral meristem-identity genes that confer identity on developing floral primordia (Weigel et al., 1992). The *LFY*, *AP1*, *CAULFLOWER* (*CAL*) and *AP2* genes appear to mutually reinforce each other, leading to a sharp transition from vegetative to reproductive development (Ferrándiz et al., 2000). In addition, *AP1*, *AGAMOUS LIKE24* (*AGL24*) and *SHORT VEGETATIVE PHASE* (*SVP*) act redundantly to control the identity of the floral meristem and to repress expression of class B, C, and E genes in *Arabidopsis* (Gregis et al., 2009). Genetic evidence suggests that *SUPPRESSOR OF CONSTANS 1* (*SOC1*) and *FLOWERING LOCUS T* (*FT*) function are closely associated with the activation of *AP1* (Teper-Bamnolker and Samach, 2005; Lee and Lee, 2010). Some results strongly show that not only *SOC1*, but also *AP1* can activate *LFY* (Liljegren et al., 1999; Lee and Lee, 2010). *TERMINAL FLOWER 1* (*TFL1*) is involved in the maintenance of the inflorescence meristem by preventing the expression of floral meristem identity genes such as *AP1* and *LFY* in the shoot apical meristem, which in turn is negatively regulated by *LFY* (Irish and Sussex, 1990; Schultz and Haughn, 1991; Weigel et al., 1992; Bowman et al., 1993). Moreover, *TFL1* function is compromised by constitutive *AP1* activity (Liljegren et al., 1999).

Extensive duplications in Poaceae resulted in the expansion and diversification of gene families. Duplications of MADS-box genes have contributed to understanding of the origin and evolution of developmental mechanisms in plant (Alvarez-Buylla et al., 2000a; Shan et al., 2009). Variance in gene family sizes occurred in a number of families in bamboo. *P. edulis* underwent a whole-genome duplication (WGD) event, which resulted in 5,370 gene losses (28% of the total genes in the collinear regions) in comparison to rice (Peng et al., 2013). In addition, some genes displayed expression subfunctionalization; for example, the genes in flowering promotion pathways (the photoperiod, gibberellin, ambient-temperature pathways) and floral pathway integrator (FPI) genes (Ehrenreich et al., 2009; Fornara et al., 2010) were not highly expressed in bamboo floral tissues. Low expression of FPI genes, which are involved in floral meristem identity, could signify that the flowering promotion pathways in bamboo may be different.

In this study, we performed a comprehensive identification and phylogenetic analysis of the MADS-box gene family in *P. eduli*s. A total of 34 MADS-box genes were identified and subjected to phylogenetic, gene structure, and domain analyses. We also studied the expression patterns of *P. eduli*s MADS-box genes under normal and abiotic stress conditions. Furthermore, expression profiles and anatomical expression were generated to screen candidate genes involved in flower development and the floral transition. The function of one of these genes, *PheMADS15*, an *AP1*-like gene, was also characterized in transgenic *Arabidopsis*. This work provides useful information on the function of this important family of transcription factors in *P. edulis*, and with both a genome sequence and a transcriptome, future systematic studies can evaluate structure-function relationships.

## Materials and Methods

### Database Searches for the Identification of MADS-Box Family Members in *P. edulis*

MADS-box protein sequences of *Arabidopsis* and *O. sativa* have been obtained from TAIR (The *Arabidopsis* Information Resource^1^) and Rice Genome Annotation Project^2^, respectively. *P. eduli*s MADS-box protein sequences were collected from Bamboo Genome Database^3^ and the accession numbers are shown in Supplementary Table S1.

The MADS-box domains were predicted through Hidden Markov Model (HMM) and redundant sequences were removed using the protein alignments with ClustalX 1.83 (Thompson et al., 1997). Information of ID accession numbers, ORF length, amino acids number, molecular weight, and isoelectric point of each protein is provided in Supplementary Table S1. For all MADS-box genes, ExPASY^4^ was employed to find the molecular weight and PI of each protein, as they were not available in the Bamboo Genome Database.

### Phylogenetic Analysis

Multiple sequence alignments of MADS-box full-length proteins were performed using ClustalX 1.83^5^ (Thompson et al., 1997). The un-rooted neighbor-joining method (Saitou and Nei, 1987) was used to construct the phylogenetic tree in MEGA 6.0^6^ (Tamura et al., 2013) software with 1000 bootstrap replicates.

### Conserved Motif and Gene Structure Analysis

Multiple EM for Motif Elicitation (MEME) version 4.9.1^7^ (Bailey and Elkan, 1995) was used to identify conserved motifs in candidate sequences with following parameters: number of repetitions = any, maximum number of motifs = 20, minimum width ≥ 6, and maximum width ≤ 200.

The MADS-box full-length cDNA sequences and the corresponding genomic DNA were collected from Bamboo Genome Database^8^. The Gene Structure Display Server (GSDS^9^) (Guo et al., 2007) was employed to identify information on intron/exon structure of the MADS-box genes.

### Calculation of *K_a_*/*K_s_* Values and Divergence Times Estimation

Alignment of nucleotide sequences of *P. eduli*s MADS-box gene pairs were aligned with ClustalX 1.83, respectively. The DNAsp5 software was used to calculate the synonymous substitution (*K_s_*) and non-synonymous substitution (*K_a_*) rates. *K_a_*/*K_s_* values were used to estimate the two types of substitutions events. The *K_s_* value was calculated for each of the MADS-box gene pairs and then used to calculate the divergence time of the duplication event (*T* = *K_s_*/2λ) using the formula: *T* = *K_s_*/2λ (Lynch and Conery, 2000), with the divergence rate λ = 6.5 × 10^-9^ (Peng et al., 2013).

### Plant Material

*Arabidopsis* plants were grown under long daylight exposure (16 h light/8 h dark) in light growth incubator maintained at 23°C with 40 to 50% humidity, and an irradiance of approximately 118 μmol m^-2^ s^-1^.

The flower buds and flower of *P. eduli*s at different flowering developmental stages were collected in Dajing County, Guilin (E 110°17′-110°47′; N 25°04′-25°48′) in Guangxi Zhuang Autonomous Region from April to August, 2012. Flower development was distinguished by four phases: the floral bud formation stage, the inflorescence growing stage, the bloom stage, the embryo formation stage (Gao et al., 2014).

### Expression Profile Analysis

Reads per kilobase of exon model per million mapped reads (RPKM) values of flowering tissues at floral organ development and shoot growth were imported into Cluster 3.0 (de Hoon et al., 2004) for windows and Java TreeView (Saldanha, 2004) to generate the heat maps. RPKM values were shown in Supplementary Table S4.

### Quantitative Real-Time PCR (QPCR)

Total RNA was extracted using the Trizol reagent (Invitrogen, USA). The quality and purified RNA was initially assessed on an agarose gel and NanoDrop 8000 spectrophotometer (NanoDrop, Thermo Scientific, Germany), and then the integrity of RNA samples was further evaluated using an Agilent 2100 Bioanalyzer (Agilent Technologies, USA). For qPCR, Primer 3 Input (version 4.0) was used to design the specific primers according to the MADS-box gene sequences. Detailed descriptions were provided in Supplementary Table S5. Data acquisition and analyses were performed by the Roche Light Cycler software.

### Subcellular Localization and Transcriptional Activation

The subcellular localization of PheMADS15 was performed by transfecting GFP-tagged PheMADS15 into rice stem and sheath protoplasts (Zhang et al., 2011). The full-length cDNA of *PheMADS15* was fused in frame with the GFP cDNA and ligated between the CaMV 35S promoter and the nopaline synthase terminator. The fluorescence signals in transfected protoplasts were examined using a confocal laser scanning microscope (Leica Microsystems).

The transcriptional activation activity of PheMADS15 was tested by transforming the pGBKT7 construct containing a fusion of PheMADS15 and the GAL4 DNA-binding domain into the yeast strain PJ69-4a. The yeast strain contains the *His-3* and *LacZ* reporter genes. The transformed yeast cells were grown on synthetic defined plates with or without *His* and assayed for β-galactosidase activity.

### *In Situ* Hybridization

RNA hybridization and immunological detection of the hybridized probes were performed based on the protocol described previously (de Almeida Engler et al., 2001). The specific probes of *PheMADS15*, *PheMADS4-1*, *PheMADS3*, *PheMADS21*, and *PheMADS5* were designed and synthesized by GENEWIZ (Supplementary Table S6). Images were obtained using the Olympus Nikon E600 microscope.

### Overexpression

The 35S:PheMADS15 sequence was amplified using specific primers (forward, 5′-GGTACCATGGGGCGCGGGAAGGTG-3′; reverse, 5′-CCCAAGCTTTCATGAAGGACGAGGAAGAGTCTG-3′) by RT-PCR with 2 μl cDNA from leaves of *P. edulis*. The product was initially cloned into pGEM-T Easy vector and then 35S:pCAMBIA2300 vector. The 35S:PheMADS15 construct was introduced into wild-type *Arabidopsis* plants (Columbia-0) through *Agrobacterium*-mediated transformation (Feldmann and Marks, 1987).

## Results

### Identification and Phylogenetic Analysis of *P. edulis* MADS-Box Genes

A total of 34 non-redundant MADS-box genes were identified in the *P. edulis* genome using rice MADS-box domain sequences as queries. To determine the evolutionary relationship of these genes in *P. edulis* and other species, we constructed a Neighbor-Joining phylogenetic tree of MADS-box proteins from *P. edulis*, rice and *Arabidopsis*. According to the previously reports in rice and *Arabidopsis*, the proteins can be classified into five functional groups (Pařenicová et al., 2003; Arora et al., 2007).

**FIGURE 1:**
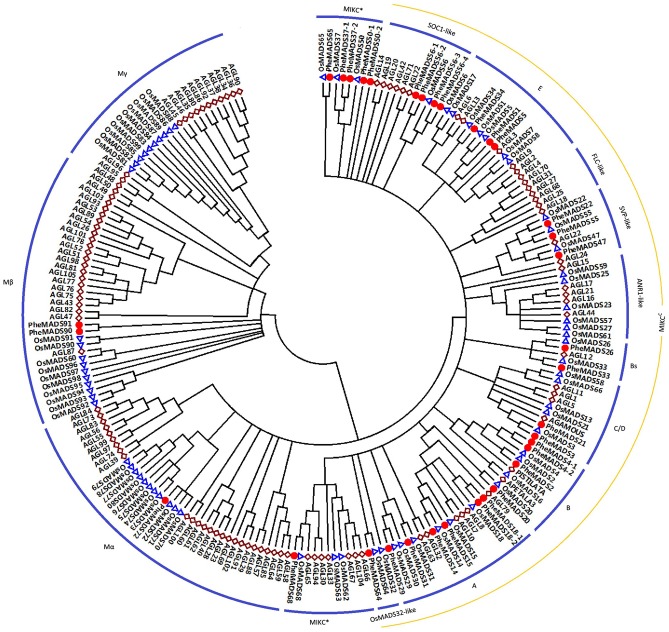
**Phylogenetic analysis of MADS-box proteins in *Phyllostachys eduli*s, rice and *Arabidopsis*.** A total of 34 MADS-box proteins in *P. eduli*s, 75 in rice and 98 in *Arabidopsis* were used to construct the NJ tree. The MADS-box proteins in *P. eduli*s were marked by red dots. Branches with less than 50% bootstrap support were collapsed.

Of the 34 identified *P. edulis* MADS-box genes, 31 grouped into the type II clade subdividing into 25 MIKC^C^-type genes and six MIKC^∗^-type genes (**Figure 1**). MIKC^C^-type genes were further divided into nine classic clades: *SOC1*-like (four genes), E (three genes), *SVP*-like (three genes), Bs (two genes), C/D (two genes), B (four genes), A (six genes), and *OsMADS32*-like (one gene). In this study, genes belonging to *FLC*-clade were absent in *P. edulis* and rice, which may be specific to *Arabidopsis*. Interestingly, *PheMADS64* was grouped with *OsMADS32*-like which is a novel monocot MADS-box gene (Sang et al., 2012). However, in contrast to previous research, the *OsMADS64* was found to cluster with *OsMADS32* to form a *OsMADS32*-like group instead of the MIKC^∗^ group (Arora et al., 2007). In the case of type I genes, including Mα and Mβ, *PheMADS90* and *PheMADS91* were identified as Mβ and *PheMADS72* grouped with Mα, but nothing grouped with the Mγ clade. Mγ, *FLC*-like and *ANR1*-like MADS-box genes were absent from *P. edulis*, indicating that these genes might have been lost after the divergence of monocots and dicots. In addition, a phylogenetic tree with bootstrap values was constructed to identify putative orthologs in *Arabidopsis*, rice and *P. edulis* using the complete protein sequences (Supplementary Table S1).

### Gene Structure and Conserved Motif Distribution Analysis

**FIGURE 2:**
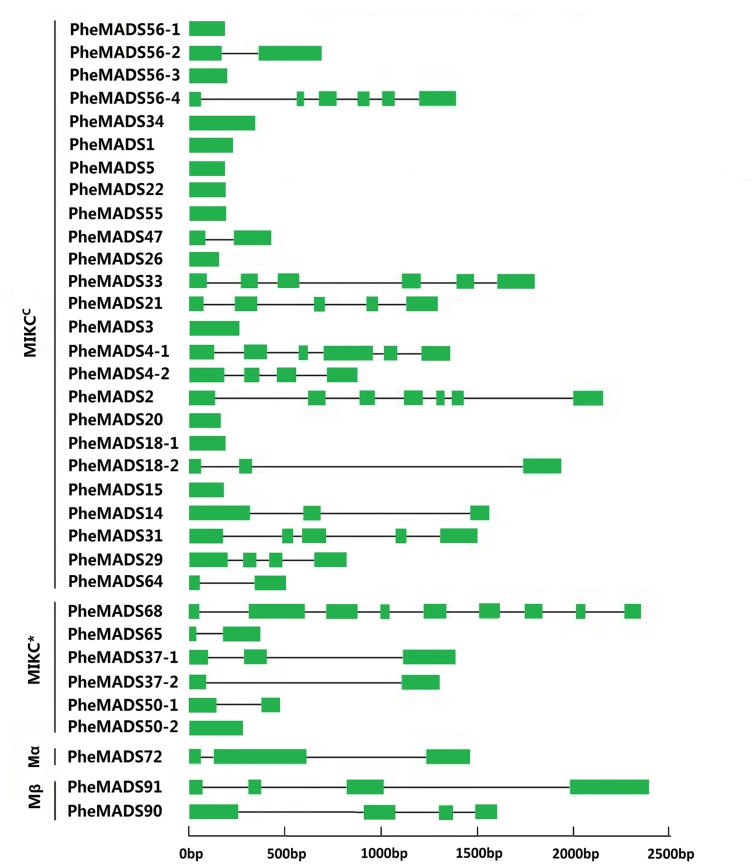
**Phylogenetic relationship and gene structure analysis of MADS-box genes in *P. eduli*s.** Un-rooted neighbor-joining tree was constructed from the alignment of full-length amino acid sequences using the MEGA5 package. Branches with less than 50% bootstrap values were collapsed. Lengths of exons and introns of each MADS-box gene were displayed proportionally. The green solid boxes represent exons; black lines represent introns.

To better understand the structural diversity of *P. edulis* MADS-box genes, intron/exon arrangements and conserved motifs were compared with phylogenetics. The MEME motif search tool and GSDS were employed to identify conserved motifs and gene structures in MADS-box genes. Intron/exon arrangements in *P. edulis* MADS-box genes were different among MIKC^C^ and MIKC^∗^ genes (**Figure 2**), similar to reports in *Arabidopsis* and rice. Nearly half of MIKC^C^ genes lacked introns, but only one MIKC^∗^ gene lacked an intron (*PheMADS50-2*). The number of introns in remaining MADS-box genes ranged from 1 to 8. The length of MADS-box proteins varied from 62 to 376 amino acids (Supplementary Table S1).

**FIGURE 3:**
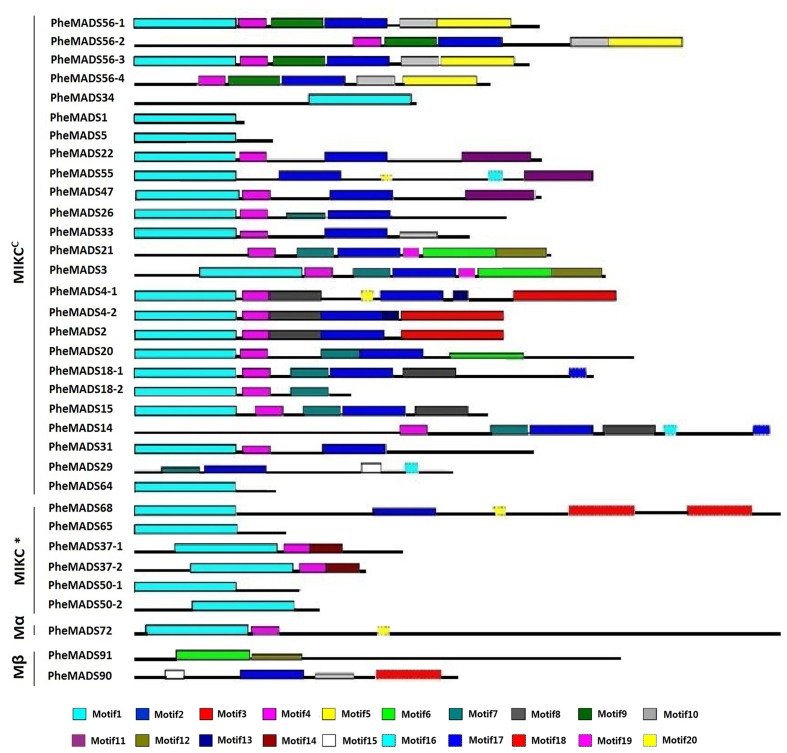
**Distribution of conserved motifs in *P. edulis* MADS-box proteins identified using MEME search tool.** Schematic representation of motifs identified in *P. edulis* MADS-box proteins using MEME motif search tool for each group given separately. Each motif is represented by a number in colored box. Length of box does not correspond to length of motif. Order of the motifs corresponds to position of motifs in individual protein sequence. For detail of motifs refer to Supplementary Material.

The MEME program was used to analyze conserved motifs in MADS-box proteins followed by SMART annotation, resulting in the identification of 20 conserved motifs (**Figure 3** and Supplementary Table S3). In all 34 *P. edulis* MADS-box proteins, excluding Mγ, most of MIKC^C^ and MIKC^∗^ groups had motif 1-type MADS domain. Motifs 2, 8, 9, and 10 were localized in the K domain. Motif 4 was also conserved across many of the MADS-box proteins, excluding Mβ, which was found in the I domain. Most of the unconserved motifs (3, 5–7, 11–20) were located in C-terminus, which is typically the most diverse region in MADS-box proteins (Kramer et al., 1998). The sequences and lengths of all the motifs were given in Supplementary Table S3.

### The Analysis of Expression Patterns of PheMADS-Box Genes during Floral Organ Development

**FIGURE 4:**
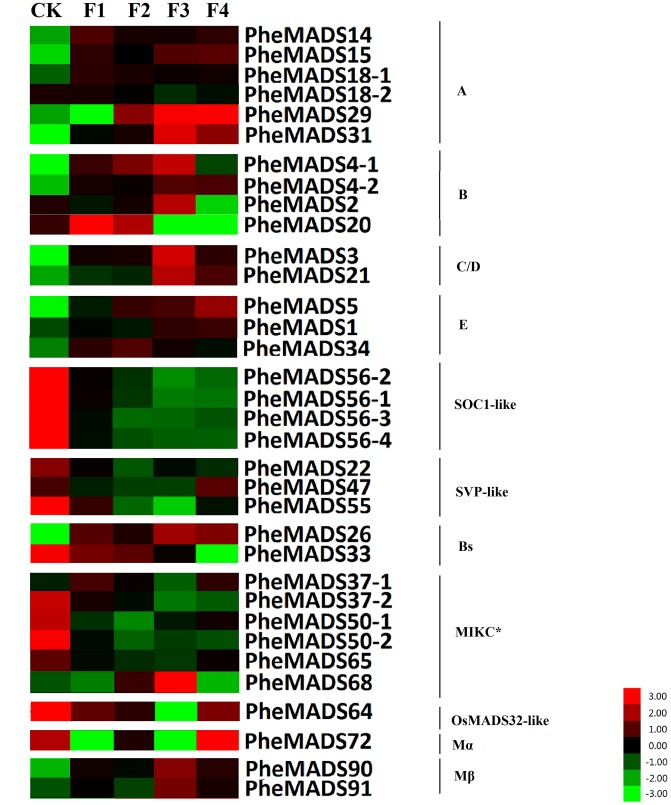
**Expression Analysis of MADS-box genes in *P. edulis*.** Hierarchical cluster display of expression profile for 34 MADS-box genes showing different expression levels in during floral organ development (CK, F1, F2, F3, and F4). Color bar at the base represents RPKM expression values, thereby green color representing low level expression, black shows medium level expression and red signifies high level expression.

MADS-box gene expression was tested at five broad categories in flowers described by Gao et al. (2014). The expression profiles were expanded by including transcriptomes from the Transcriptome Sequencing Bamboo Genome Database, including: leaves from non-flowering (CK) and four flowering developmental stages (F) of *P. edulis* (Supplementary Table S4). MADS-box genes were classified into 11 groups based on phylogenetic analysis during flowering developmental stages (**Figure 4**). The expression levels of the A and B class PheMADS genes were high in F1 and F2 and decreased through floral maturity. In contrast, the expressions of C, D, and E class PheMADS genes were reduced at the floral bud formation stage, increased at the third flowering developmental stage and embryo formation stage. Besides, *PheMADS26* (Bs-class), *PheMADS68* (MIKC^∗^-type) and *PheMADS72* (Mβ-class) were expressed predominantly at the floral formation stage.

**FIGURE 5:**
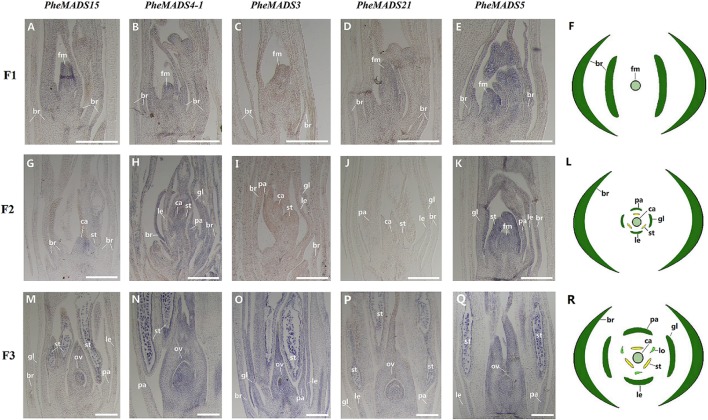
***In situ* analysis of *PheMADS15*, *PheMADS4-1*, *PheMADS3*, *PheMADS21*, and *PheMADS5*.** F1: The floral bud formation stage. F2: The inflorescence growing stage. F3: The flowers with both pistils and stamens emerging from glumes at bloom stage. A-S: *In situ* localization of *PheMADS15* **(A,G,M)**, *PheMADS4-1* **(B,H,N)**, *PheMADS3* **(C,I,O)**, *PheMADS21* **(D,J,P)** and *PheMADS5* **(E,K,Q)** transcripts from F1 to F3; floral diagrams of F1 **(F)**, F2 **(L)**, and F3 **(R)**. fm, floral meristem; br, bract; gl, glume; le, lemma; pa, palea; st, stamen; ca, capel; ov, ovule. Bars = 100 μm.

**FIGURE 6:**
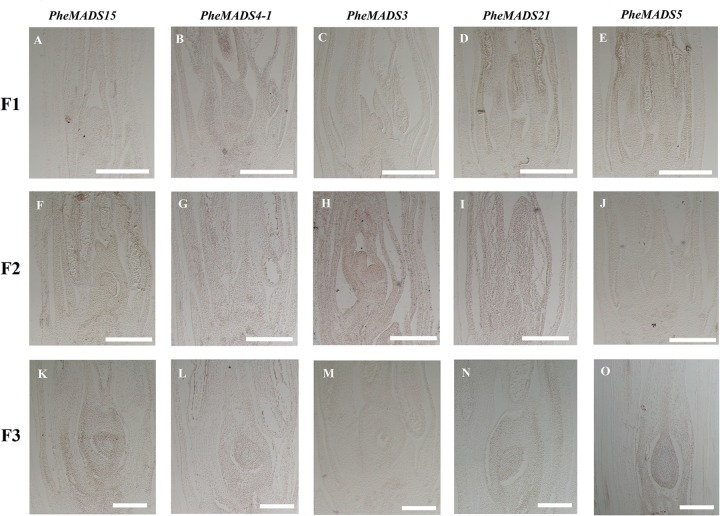
**Sense controls of *PheMADS15*, *PheMADS4-1*, *PheMADS3*, *PheMADS21*, and *PheMADS5*.** F1: The floral bud formation stage. F2: The inflorescence growing stage. F3: The flowers with both pistils and stamens emerging from glumes at bloom stage. **(A–O)** Sense controls for *PheMADS15* **(A,F,K)**, *PheMADS4-1* **(B,G,L)**, *PheMADS3* **(C,H,M)**, *PheMADS21* **(D,I,N),** and *PheMADS5* **(E,J,O)** transcripts from F1 to F3. Bars = 100 μm.

From the outer to the inner whorl within the floral organ, the *P. edulis* flower consisted of four concentric whorls comprising lemma (whorl 1), palea (whorl 1), three lodicules (whorl 2), three stamens (whorl 3) and in the center, pistil (whorl 4) in which the ovule develops (**Figures 5F,L,R** and Supplementary Figure S3). These organs together formed a floret. Our results indicated that A-class genes, *PheMADS14*, *PheMADS15*, and *PheMADS18-1* were expressed throughout, and higher at the floral bud formation stage, while *PheMADS29* and *PheMADS30* were preferentially expressed from F2 to F4 (**Figure 4**). Based on *in situ* hybridization analysis, *PheMADS15* was expressed in the early spikelet meristem, the primordia of flower organs, and the reproductive organs (**Figures 5A,G,M**). Based on the phylogenetic tree analysis, the C and D class contain *PheMADS3* and *PheMADS21* (**Figure 1**). *PheMADS3* and *PheMADS21* were mainly expressed in stamen and pistils formation stage (**Figure 4**). In addition, the *in situ* hybridization data showed that *PheMADS3* and *PheMADS21* mRNA were highly expressed in the stamen and developing embryo (**Figures 5C,D,I,J,O,P**). These data were consistent with those of *PheMADS3* and *PheMADS21* from RNA-seq. The E class genes in the *SEP* lineage in *P. edulis* were *PheMADS1*, *PheMADS5*, and *PheMADS3*4. *PheMADS1* and *PheMADS5* were highly expressed in the third flowering developmental stage and embryo formation stage (**Figure 4**). The spatial and temporal expression patterns *PheMADS5* were detected from the early floral bud to the maturely floral organ by *in situ* hybridization in *P. edulis* (**Figures 5E,K,Q**). However, *PheMADS34* was expressed predominantly at the floral bud formation stage and declined during floral development. In addition, the functionally characterized MADS-box genes of rice and *Arabidopsis* are listed in Supplementary Table S2 which provided support toward ABCDE model. Sense controls for five MADS-box genes are in **Figure 6**.

On the contrary, the expression of the remaining genes of *PheMADSs* was lower than that of ABCDE PheMADS genes in *P. edulis* floral development. However, *PheMADS26* (Bs-class), *PheMADS68* (MIKC^∗^-type) and *PheMADS72* (Mβ-class) were expressed predominantly at the floral formation stage (**Figure 4**). Perhaps these genes might be also involved in the development of flower organs.

**FIGURE 7:**
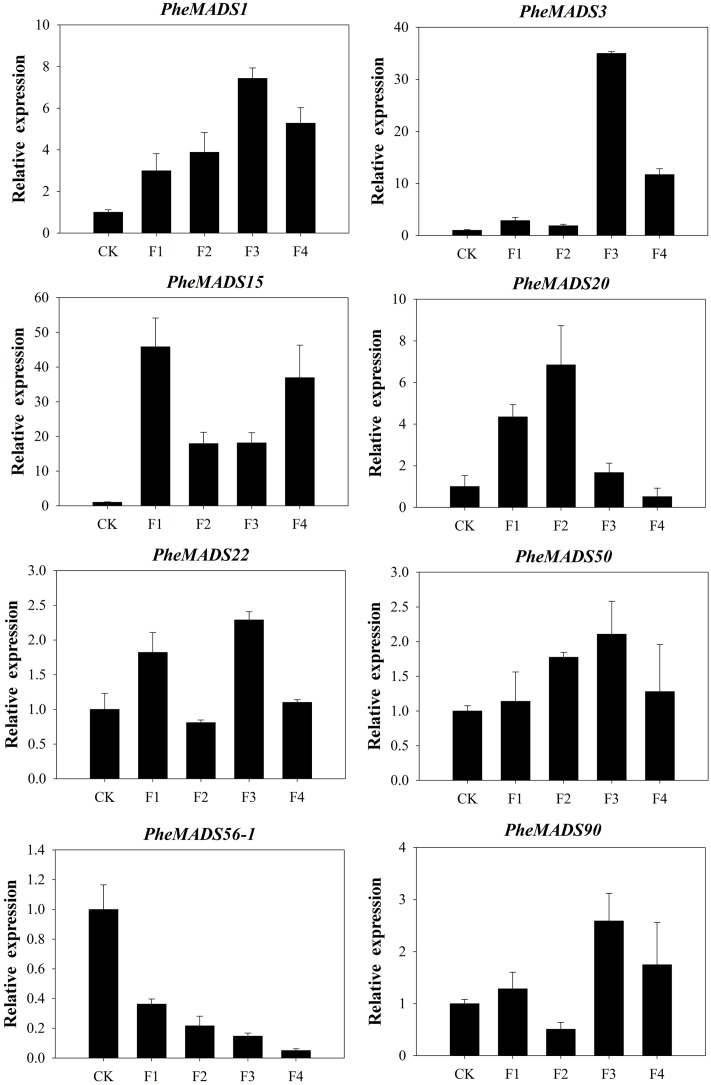
**The expression profiles of eight selected genes from flowering tissues in different flower developmental stages and leaves of non-flowering plants (CK).** The transcript levels were normalized to that of TIP41 (Fan et al., 2013), and the level of each gene in the control was set at 1.0. Error bars represented the SD for three independent experiments.

To confirm that *PheMADS* genes from RNA-seq are expressed, eight genes were selected for validation by qPCR (**Figure 7**). The expression of two *PheMADS* genes (*PheMADS3* and *15*) were up-regulated in floral tissues 10-fold more than non-flowering leaves. However, the expression of *PheMADS56-1* decreased significantly during flower development. According to the qPCR results, the expression patterns for all eight genes from qPCR were similar to that obtained from the Illumina analysis, thus strengthening the reliability of the RNA-seq data.

### Duplication and Functional Divergence of MADS-Box Gene Pairs in *P. edulis*

A significant role for gene duplication in the proliferation and evolution of biological complexity of MADS-box genes has been postulated in many divergent plant species (Alvarez-Buylla et al., 2000b; Shan et al., 2009). Most duplicated genes diverge to compartmentalize function (sub-functionalization) or gain novel function (neo-functionalization), and can increase biological complexity (Lynch and Conery, 2000; Ohno, 2013). A rate of 6.5 × 10^-9^ substitution per synonymous site per year was used to calculate the divergence time between 13 pairs of closely related MADS-box genes in a phylogenetic tree (Gaut et al., 1996). The divergence for most PheMADS-box gene pairs is around 10 to 30 MYA (Million Years Ago) (**Table 1**), which is a similar time fram as the *P. edulis* WGD event (Peng et al., 2013), which occurred later than *Brachypodium* at ∼70 MYA (Wei et al., 2014). In contrast, six gene pairs (*PheMADS50-1/50-2*, *1/5*, *14/15*, *64/65*, *90/91*, and *26/33*) diverged 31 to 119 MYA, which does not correlate with the *P. edulis* WGD.

**Table 1:** Estimated divergence period of MADS-box gene pairs in *Phyllostachys edulis.*

Gene pairs			*K_s_*	*K_a_*	*K_a_/K*_s_	MYA
PheMADS37-1	vs.	PheMADS37-2	0.1375	0.0499	0.3629	10.58
PheMADS4-1	vs.	PheMADS4-2	0.143	0.0176	0.1230	11
PheMADS56-3	vs.	PheMADS56-4	0.2129	0.1504	0.7064	16.38
PheMADS3	vs.	PheMADS21	0.2468	0.192	0.7779	18.98
PheMADS18-1	vs.	PheMADS18-2	0.2528	0.0839	0.3319	19.45
PheMADS56-1	vs.	PheMADS56-2	0.2794	0.2123	0.7598	21.49
PheMADS22	vs.	PheMADS55	0.3963	0.2558	0.6455	30.48
PheMADS50-1	vs.	PheMADS50-2	0.4059	0.1858	0.4578	31.22
PheMADS1	vs.	PheMADS5	0.4651	0.0442	0.0950	35.78
PheMADS14	vs.	PheMADS15	0.4727	0.2265	0.4792	36.36
PheMADS64	vs.	PheMADS65	0.5772	0.3373	0.5844	44.4
PheMADS90	vs.	PheMADS91	1.1226	0.9097	0.8104	86.35
PheMADS26	vs.	PheMADS33	1.5552	0.4713	0.3030	119.63


**K_s_*, synonymous substitution rate; *K_a_*, non-synonymous substitution rate; MYA, million years ago.*

A *K_a_/K*_s_ ratio less than 1 is indicative of purifying selection and a ratio greater than 1 is indicative of diversifying selection. With pairwise comparisons we found that for all 13 PheMADS-box gene pairs evolved under purifying selection (**Table 1**). Interestingly, further analyses indicated that some closely related gene pairs had different expression patterns and subtle functional divergence. Most notably, for MIKC^∗^-type, *PheMADS37-1* expressed predominantly during floral development, whereas *PheMADS37-2* expression was detectable in leaves from non-flowering plants. These results indicated that PheMADS-box genes diverged in function whilst also undergoing strong purifying selection.

### Identification and Sequence Analysis of the *PheMADS15* Gene

To elucidate the role of *PheMADS15* in flower formation in *P. edulis*, we identified the *PheMADS15* cDNA encoding a highly conserved MADS domain. *PheMADS15* appeared to be a full-length cDNA of 630 bp encoding a protein of 209 amino acids residues (Supplementary Figure S2).

**FIGURE 8:**
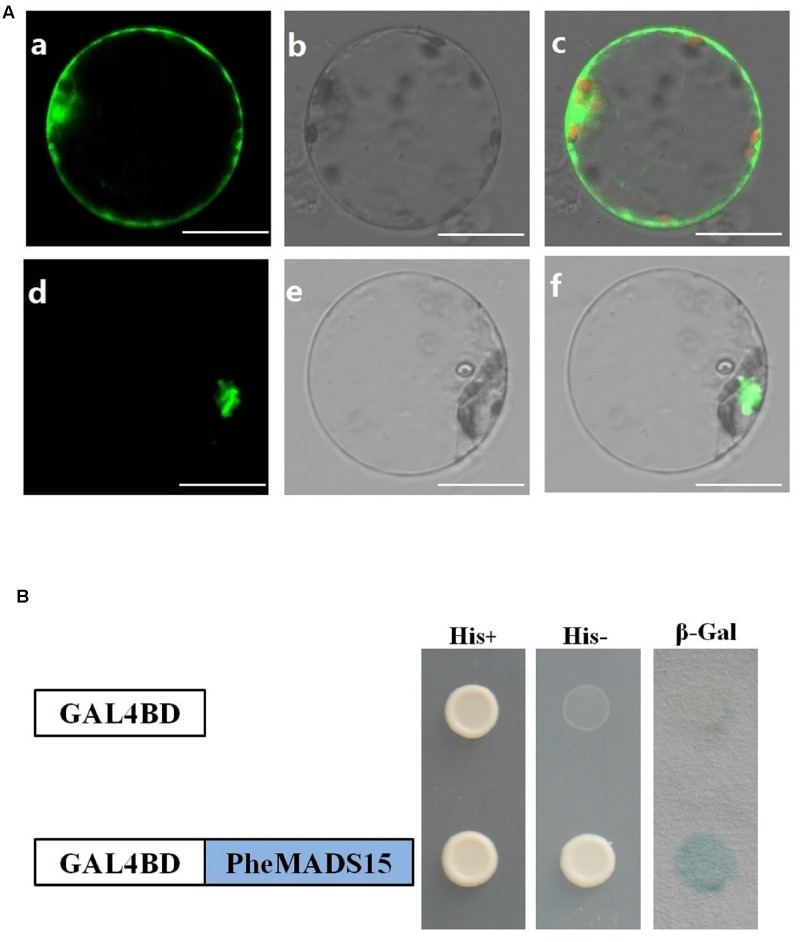
**Subcellular localization and transcriptional activation analysis of PheMADS15.** **(A)** (a–c), rice protoplasts expressing 35S-GFP, (d–f), rice protoplasts expressing PheMADS15-GFP. Bar = 10 μm. **(B)** Transcriptional activation analysis of full-length PheMADS15 fused with the GAL4 DNA binding domain in yeast showing its ability to activate the expression of the *His-3* and β-Gal reporter genes.

While green fluorescent protein (GFP) alone exhibited a dispersed cytoplasmic distribution, GFP tagged *PheMADS15* was indeed located in the nucleus, in accordance with its function as a transcription factor (**Figure 8A**). In addition, we fused PheMADS15 with the GAL4 DNA-binding domain (GAL4DB) and tested its ability in a yeast reporter construct. PheMADS15 was able to activate the expression of the *His-3* and β*-Gal* reporter gene (**Figure 8B**). *PheMADS15* was highly expressed in early blooming stages (**Figure 5A**), closely followed by later blooming stages, but just above detection in leaf samples. These results indicated that *PheMADS15* might play an important role in flower formation at an early stage, as a transcription factor.

### Overexpression of *PheMADS15* in *Arabidopsis* Plants (Wild-Type) Promotes Flowering Time

**FIGURE 9:**
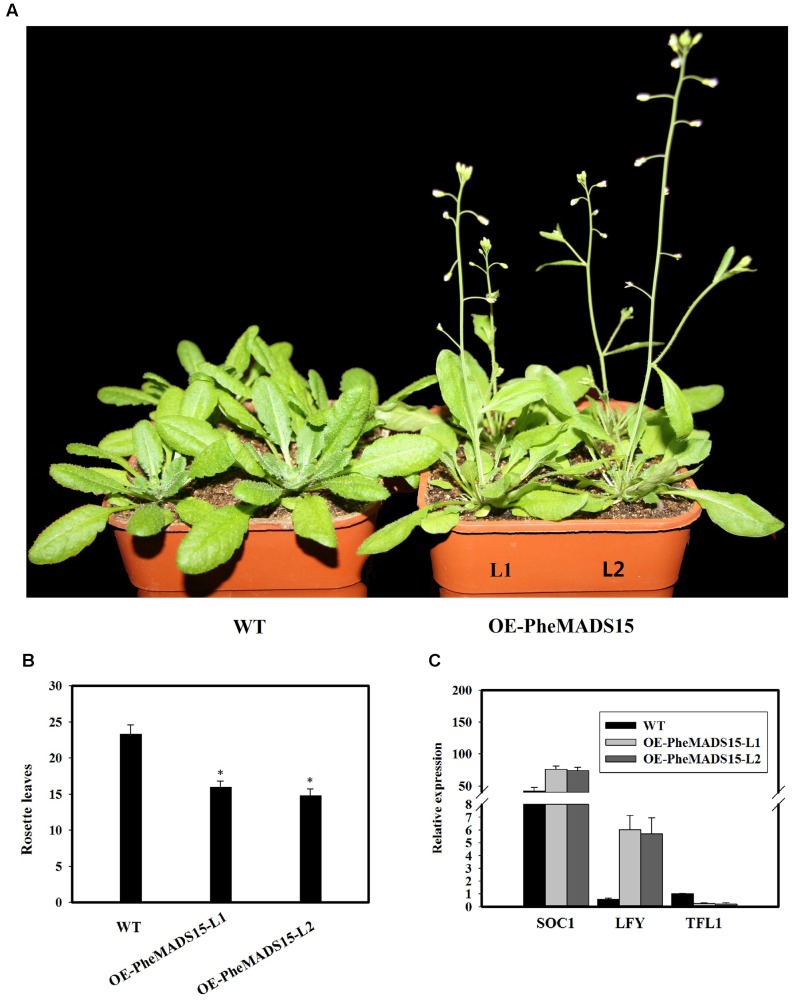
**Analysis of an early flowering phenotype by overexpression of *PheMADS15* in *Arabidopsis*.** **(A)** The flowering phenotype of wild-type (WT), OEPheMADS15-L1 and OEPheMADS15-L2 grown for 4 weeks at 23°C under long-day conditions. **(B)** Flowering time scored as the number of rosette leaves at flowering of wild-type and transgenic plants at 23°C under long-day conditions. **(C)** Transcript levels of *SOC1*, *LFY*, and *TFL1* in wild-type and transgenic plants (L1 and L2) were evaluated by qPCR. *Arabidopsis* β-tubulin expression was used as a control. Total RNA from 5-week-old whole- *Arabidopsis* tissues, including leaves and shoot apex, were used for *PheMADS15*, *SOC1*, *LFY*, and *TFL1* examination. Error bars indicate standard deviations. Asterisks indicate a statistically significant difference between wild-type and transgenic plants (*P* < 0.05 by student’s *t*-test).

To further investigate the role of *PheMADS15* in the transcriptional regulation of flowering time, *PheMADS15* was overexpressed in *Arabidopsis* (WT). At least 54 transgenic plants expressing *PheMADS15* were generated and examined for their morphology in the T_1_ generation (Supplementary Figure S1). The overexpressed plants showed an early flowering phenotype (**Figures 9A,B**). We further investigated the expression of *SOC1*, *LFY*, and *TFL1* in the T_3_ generation to ascertain the downstream effects of this construct (**Figure 9C**). *SOC1* and *LFY* had a dramatic expression increases, while *TFL1* expression was rather low in compared to wild type (**Figure 9C**), which was a similar phenomenon exhibited by overexpression of *Arabidopsis AP1* (Liljegren et al., 1999).

## Discussion

### The Slow Birth and Death Rate for MADS-Box Genes of *P. edulis*

The MADS-box gene family in plants has expanded though gene duplication events owing to multiple whole genome duplication events in many plants (Gaut, 2002; Paterson et al., 2004; Yu et al., 2005). Most of the Type II MADS-box genes that mainly control flower development were generally associated with some whole genome duplication events (Causier et al., 2005). On the contrary, the duplications inducing more Type I MADS-box genes can be attributed to smaller scale local duplication events (Nam et al., 2004). We found that *P. edulis* had a comparable number of MADS-box genes in type II group, but significantly fewer of Mα and Mβ genes than rice and *Arabidopsis*, indicating that *P. edulis* genome experienced tandem duplications (Vogel et al., 1992; Guo et al., 2004). Six pairs (*PheMADS37-1/37-2*, *PheMADS4-1/4-2*, *PheMADS56-3/56-4*, *PheMADS3/21*, *PheMADS18-1/18-2*, and *PheMADS56-1/56-2*) in *P. eduli*s had very consistent divergence times, suggesting that these gene pairs followed the WGD event of *P. eduli*s. However, for Mβ, *PheMADS90/91* divergence was estimated at about 86 MYA and represented a anciently duplicated gene pair, indicating a smaller scale local duplication event. Thus, for *P. edulis*, fewer duplication events led to a slower birth and death rate after bamboo diverged from other grasses (Peng et al., 2013).

### ABCDE Genes Have Important Functional Conservation and Diversification among *P. edulis*, Rice and *Arabidopsis*

MADS-box genes have been found to evolve through neofunctionalization or subfunctionalization after gene duplication events (Irish and Litt, 2005). Moreover, we found that homologous MADS-box genes had different expression profiles, which offered some evidence about functional divergence occurring after the divergence of *P. edulis*, rice and *Arabidopsis* (Vogel et al., 1992).

In *Arabidopsis*, *AP1* played an important role in the determination of the identity of sepals and petals and furthermore specifies floral meristem identity (Kater et al., 2006). The *AP1* homolog *OsMADS14* was highly expressed in inflorescence and caryopses through transcript analysis (Pelucchi et al., 2002). Besides, *OsMADS15* and *OsMADS18* were activated in the meristem at phase transition in rice (Kobayashi et al., 2012). In *Bambusa edulis*, as the A class gene, *BeMADS14* was expressed throughout, but higher in the lemma and pistil, *BeMADS15* was detected in the lemma and palea (Shih et al., 2014). Based on RNA-seq analysis, *PheMADS14* showed a similar expression pattern, but very low expression in floral organs differentiation stage (**Figure 4**). Meanwhile *PheMADS15* mRNA obviously accumulated in the meristem at phase transition by *in situ* hybridization. These data showed that *PheMADS15* was involved in flower bud differentiation. The expression pattern of *PheMADS18-1* and *PheMADS18-2* which were detected in flower bud formation, was different from *OsMADS18* with high expression in leaves following germination (Fornara et al., 2004). In *P. edulis*, *PheMADS29* and *PheMADS31* were mainly expressed in mature organs and developing caryopses. These data were consistent with that of *OsMADS29*, which was expressed in seed development of rice (Yang et al., 2012). Our results showed that five *AP1*-like genes were uniformly expressed in *P. edulis* floral organs. This similar expression pattern in floral organs was also shown for *AP1-*like genes in *Arabidopsis* (Mandel et al., 1992) and rice (Arora et al., 2007).

In *Arabidopsis*, *AP3* and *PI*, two class B floral organ identity genes, belonged to the *DEF*-like and *GLO*-like gene groups, respectively (Jack et al., 1992; Goto and Meyerowitz, 1994). Rice *in situ* hybridization data showed that two *PI* homologs, *OsMADS2* and *OsMADS4* played important roles in lodicule and stamen development (Yao et al., 2008). Whether of *PI* or *AP3* lineage, the mRNA of B class genes (*PheMADS2*, *PheMADS4-1* and *PheMADS4-2*) showed a similar expression pattern: mainly in stamen development (F3) (**Figure 4**). Rice *OsMADS16*/*SPW1* and maize *SILKY1* (*SL*) mRNA were detected mainly in the lodicules and stamen primordia during floral development, but not in developing carpels (Ambrose et al., 2000; Nagasawa et al., 2003). In wheat, the expression of *TaAP3* was obviously accumulated in mature female organs, but the function of *TaAP3* was unknown (Paollacci et al., 2007). To further explore the spatial and temporal expression pattern of B class genes, a stronger expression of *PheMADS4-1* was observed in stamen by *in situ* hybridization (**Figures 5B,H,N**). For *B. edulis*, *BeMADS2*, the *PI*/*GLO*-like gene, also displayed similar expression patterns with *PheMADS4-1*, was highly expressed in anthers (Shih et al., 2014). This result correlated with that of *PI* and *AP3*. However, only *PheMADS20* was strongly expressed in the spikelet primordia before lemma and palea initiation (F1) (**Figure 4**).

In *Arabidopsis*, *AG* was a typical class C gene (Yanofsky et al., 1990). As proposed by the ABC model, the *AG* gene was essential to specify stamen and carpel identity and floral determinacy. In rice, analysis of *osmads3 osmads58* double mutant revealed the fact that *OsMADS3* and *OsMADS58* were involved in reproductive organ identity and accumulation of lodicules in the whorl 3 and whorl 4 (Dreni et al., 2011). Besides, in wheat, *TaAG-1* and *TaAG-2* transcripts were highly expressed in the stamens and pistils (Paollacci et al., 2007). *PheMADS3*, was also detected in stamens, carpels and ovule primordial by *in situ* hybridization (**Figure 5O**). *PheMADS21* was also part of the *AG*-lineage and mainly expressed in anthers and pistils, with especially high levels in anthers by *in situ* hybridization (**Figure 5P**). In *Arabidopsis*, the class D gene, *STK*, was exclusively expressed in ovules (Pinyopich et al., 2003). In rice, two class D genes have been identified, namely *OsMADS13* (Lopez-Dee et al., 1999) and *OsMADS21* (Lee et al., 2003) based on phylogenetic reconstruction. The expression pattern of *OsMADS13* which was specifically expressed in the ovule was very similar to maize *ZAG2* and *Arabidopsis STK* (Schmidt et al., 1993; Lopez-Dee et al., 1999; Dreni et al., 2007). Moreover, the expression region of *OsMADS21* which was highly expressed in the inner cell layers of the ovary and in the ovule integuments, overlapped with that of *OsMADS13* (Dreni et al., 2007). The expression pattern of *PheMADS21* was slightly different from the *Arabidopsis* ortholog *STK* and the rice ortholog *OsMADS21*. Thus, much deeper investigations are needed to further substantiate the classification and functioning of *PheMADS3* and *PheMADS21*.

In *Arabidopsis*, the E class genes, such as *Arabidopsis SEP* genes were involved in the specification of sepal, petal, stamen, carpel, and ovule identity and interact with the class A, B, C, and D genes to form higher order MADS-box protein complexes (Honma and Goto, 2001; Pelaz et al., 2001; Favaro et al., 2003). In *P. edulis*, three E class genes, such as *PheMADS1*, *PheMADS5*, and *PheMADS34* belonged to the *SEP* lineage. On *in situ* hybridization analysis, *PheMADS5* was highly expressed throughout the floral meristem and subsequently detected in palea, lemma, and anthers in the mature flower (**Figures 5E,K,Q**). In rice, *OsMADS5*, a *SEP-*like gene, caused homeotic transformation of all floral organs except the lemma into leaf-like organs (Cui et al., 2010). The expression of *PheMADS34* which was high in flower bud formation, was similar to *OsMADS34.* However, *OsMADS34* played a key role in lemma/palea, lodicules, stamens, and carpel (Gao et al., 2010). These findings show many significant differences can be observed between rice and moso bamboo. Future functional studies will have to explore biological function of these *PheMADS* genes.

### Overexpression of *PheMADS15* Promotes Flowering Time

In rice, *OsMADS14* and *OsMADS15* were previously identified as flowering regulators (Kim et al., 2007; Lu et al., 2012). Here, we report the identification and characterization of a MADS-box gene from *P. edulis*, *PheMADS15*, which through ectopic overexpression triggered earlier flowering time in *Arabidopsis. PheMADS15*, an *AP1*-like gene, is highly expressed during flower bud morphological differentiation (**Figure 5A**) and it is located in the nucleus (**Figure 8B**). *LFY* and *AP1* which were expressed in the converted floral meristems primarily control *Arabidopsis* flower meristems (Shannon and Meeks-Wagner, 1991; Weigel et al., 1992; Ferrándiz et al., 2000). In this study, early flowering time was also observed in *35S*: *PheMADS15* transgenic *Arabidopsis*. Meanwhile, the expression level of *LFY* and *SOC1* was up-regulated in *35S*:*PheMADS15* transgenic *Arabidopsis* compared with wild type *Arabidopsis. SOC1* promoted floral transitions and is considered as one of the core regulators of flowering in *Arabidopsis* (Moon et al., 2003; Liu et al., 2008; Lee and Lee, 2010; Dorca-Fornell et al., 2011). *AP1* is another positive regulator of *LFY* and is highly expressed in converted floral meristems (Liljegren et al., 1999). In addition, *OsMADS14*, *OsMADS15*, and *OsMADS18*, three *AP1/FUL*-like genes, were involved in the regulation of flowering time (Kobayashi et al., 2012). This leads us to suspect that *PheMADS15* promotes flowering time by regulating *LFY* and *SOC1* directly or indirectly. This is consistent with the previous reports about some *AP1-*like genes (Weigel et al., 1992), suggesting that *PheMADS15* is a functional ortholog of *Arabidopsis AP1*. Further research on the transcriptional regulatory network mediated by PheMADSs will increase knowledge surrounding the transcriptional regulation of flowering time in *P. edulis*.

## Author Contributions

ZC, WG, and LL performed all the experiments, data analysis and wrote the paper. XL and SM analyzed data. DH, YM, JL, and QB revised the manuscript. JG designed and supervised experiments.

## Conflict of Interest Statement

The authors declare that the research was conducted in the absence of any commercial or financial relationships that could be construed as a potential conflict of interest.

## Footnotes

1 http://www.arabidopsis.org/

2 http://rice.plantbiology.msu.edu/

3 http://202.127.18.221/bamboo/down.php

4 http://web.expasy.org/compute_pi/

5 http://www.clustal.org/

6 http://www.megasoftware.net/mega.html

7 http://alternate.meme-suite.org/tools/meme

8 http://www.bamboogdb.com/

9 http://gsds.cbi.pku.edu.cn/
