# Supplementary material for: Analysis of MADS-Box Gene Family Reveals Conservation in Floral Organ ABCDE Model of Moso Bamboo (Phyllostachys edulis)
Source: Front Plant Sci. 2017 May 3;8:656. doi: 10.3389/fpls.2017.00656 (PMC5413564; doi:10.3389/fpls.2017.00656)
Supplement: Supplementary file 1 [file Data_Sheet_1.DOC]

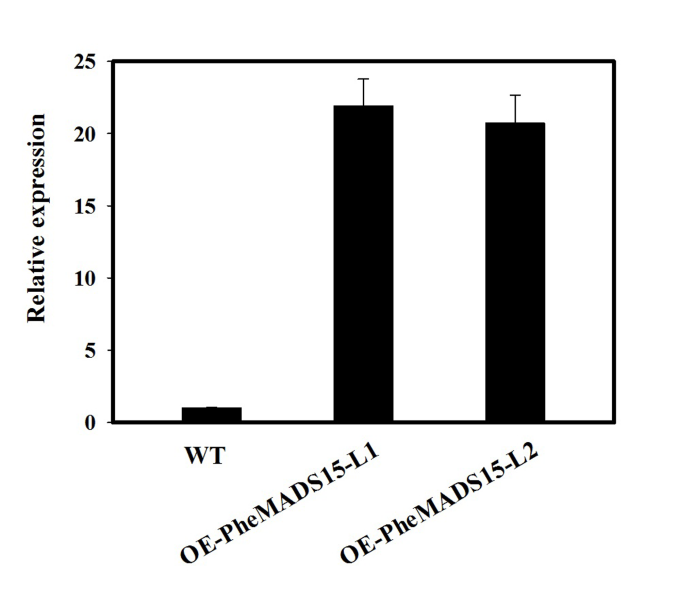


**Figure S1** The detection of *PheMADS15* expression in *35S:PheMADS15* transgenic *Arabidopsis* plants. Transcript levels of *PheMADS15* in wild-type and transgenic plants L1(Line-1) and L2 (Line-2) were elaluated by qPCR. Wild-type and transgenic plants (Line-1 and Line-2 ) grown for four weeks at 23ºC under long-day conditions.


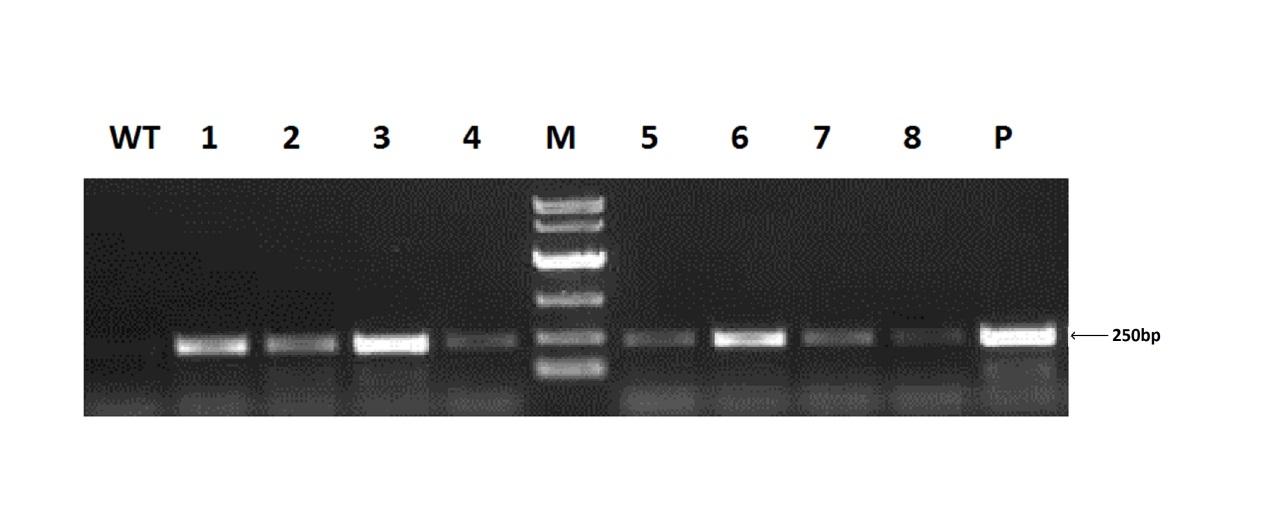


**Figure S2** PCR analysis of transgenic *Arabidopsis* plants. Genomic DNAs were isolated from plants transformed with the *35S:PheMADS15* vector. PCR amplification used primers specific for the production of a 250 bp fragment from part of NPTII region. M, D2000 DNA Ladder; WT, non-transgenic plants; P, corresponding plasmid DNA (positive control); Number on the right indicate DNA marker sizes in base pairs.


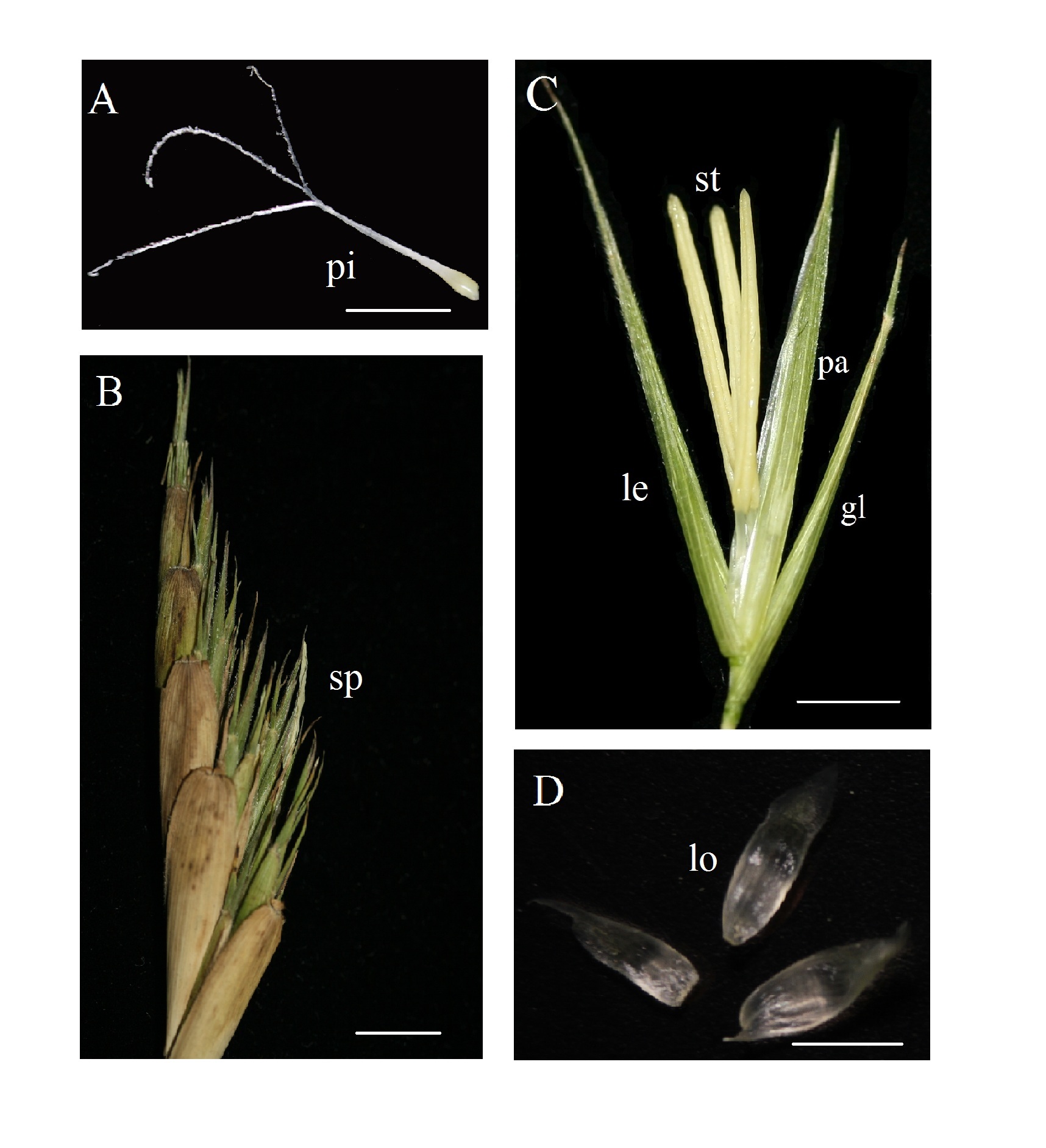


**Figure S3** *P. edulis* Wild-type flowers. A: Representatives of the spikelet. C: Representatives of stamen, glume, lemma and palea. B: Representatives of pistil (stigma, style and ovary). D: Representatives of lodicules. gl, glume; le, lemma; pa, palea; lo, lodicules; st, stamen; sp, spikelet; fb, flower bud. Bars = 5mm. *Arabidopsis* and rice wild-type flowers were reported previously (Kater et al*.*, 2006).

| Table S1 List of 34 MADS-box genes identified in *P. edulis* and their sequence characteristics | | | | | | | |
| --- | --- | --- | --- | --- | --- | --- | --- |
| Name | ID | Protein | | | PI | Intron | Type |
|  |  | ORF (bp) | Length (aa) | Mol. Wt (d) |  |  |  |
| *PheMADS1* | PH01000216G0990 | 186 | 62 | 7.14 | 10.36 | 0 | MIKC^C^ |
| *PheMADS2* | PH01000317G0080 | 627 | 209 | 24.09 | 8.76 | 6 | MIKC^C^ |
| *PheMADS3* | PH01002127G0260 | 795 | 265 | 30.06 | 8.91 | 0 | MIKC^C^ |
| *PheMADS4-1* | PH01000794G0210 | 816 | 272 | 32.1 | 8.62 | 5 | MIKC^C^ |
| *PheMADS4-2* | PH01001878G0200 | 627 | 209 | 24.53 | 8.48 | 3 | MIKC^C^ |
| *PheMADS5* | PH01001952G0230 | 234 | 78 | 8.94 | 10.64 | 0 | MIKC^C^ |
| *PheMADS14* | PH01000222G1190 | 1074 | 358 | 38.07 | 8.16 | 2 | MIKC^C^ |
| *PheMADS15* | PH01000606G0250 | 600 | 200 | 23.48 | 8.98 | 0 | MIKC^C^ |
| *PheMADS18-1* | PH01000306G0610 | 771 | 257 | 28.99 | 9.43 | 0 | MIKC^C^ |
| *PheMADS18-2* | PH01002743G0050 | 369 | 123 | 14.2 | 9.73 | 2 | MIKC^C^ |
| *PheMADS20* | PH01000006G0210 | 840 | 280 | 31.5 | 6.47 | 0 | MIKC^C^ |
| *PheMADS21* | PH01001278G0330 | 702 | 234 | 27.07 | 7.14 | 4 | MIKC^C^ |
| *PheMADS22* | PH01000437G0930 | 684 | 228 | 25.55 | 5.4 | 0 | MIKC^C^ |
| *PheMADS26* | PH01005177G0100 | 630 | 210 | 23.75 | 6.21 | 2 | MIKC^C^ |
| *PheMADS29* | PH01003236G0170 | 534 | 178 | 20.45 | 5.13 | 3 | MIKC^C^ |
| *PheMADS31* | PH01000338G0120 | 675 | 225 | 26.28 | 9.04 | 4 | MIKC^C^ |
| *PheMADS33* | PH01000616G0020 | 567 | 189 | 21.51 | 9.64 | 5 | MIKC^C^ |
| *PheMADS34* | PH01000222G1060 | 477 | 159 | 18.04 | 10.55 | 1 | MIKC^C^ |
| *PheMADS37-1* | PH01000117G1210 | 453 | 151 | 17.04 | 6.4 | 2 | MIKC^*^ |
| *PheMADS37-2* | PH01001303G0110 | 393 | 131 | 14.93 | 9.94 | 1 | MIKC^*^ |
| *PheMADS47* | PH01000038G1550 | 690 | 230 | 26.04 | 7.76 | 0 | MIKC^C^ |
| *PheMADS50-1* | PH01002152G0090 | 285 | 95 | 10.79 | 10.65 | 1 | MIKC^*^ |
| *PheMADS50-2* | PH01002755G0230 | 312 | 104 | 11.3 | 10.41 | 0 | MIKC^*^ |
| *PheMADS55* | PH01000080G0200 | 774 | 258 | 29.01 | 6.8 | 0 | MIKC^C^ |
| *PheMADS56-1* | PH01000759G0450 | 678 | 226 | 25.7 | 9.12 | 0 | MIKC^C^ |
| *PheMADS56-2* | PH01000107G0570 | 924 | 308 | 34.54 | 8.22 | 1 | MIKC^C^ |
| *PheMADS56-3* | PH01000059G1270 | 666 | 222 | 25.04 | 9.52 | 0 | MIKC^C^ |
| *PheMADS56-4* | PH01002152G0120 | 603 | 201 | 22.68 | 9.1 | 5 | MIKC^C^ |
| *PheMADS64* | PH01000518G0720 | 228 | 76 | 8.91 | 10.11 | 1 | MIKC^C^ |
| *PheMADS65* | PH01003178G0150 | 255 | 85 | 9.76 | 11.3 | 1 | MIKC^*^ |
| *PheMADS68* | PH01000466G0340 | 1107 | 369 | 41.21 | 6.19 | 8 | MIKC^*^ |
| *PheMADS72* | PH01000954G0350 | 1128 | 376 | 41.65 | 9 | 2 | Mα |
| *PheMADS90* | PH01001174G0480 | 540 | 180 | 20.25 | 5.45 | 3 | Mβ |
| *PheMADS91* | PH01001750G0200 | 819 | 273 | 30.93 | 5.91 | 3 | Mβ |

**Table S1** List of 34 MADS-box genes identified in *P. eduli*s and their sequence characteristics (bp, base pair; aa, amino acids; D, Dalton).

| Table S2 The accession numbers and characterization of MADS-box genes between rice and *Arabidopsis* | | | |
| --- | --- | --- | --- |
| Gene name | Accession numbers | Accession numbers (NCBI) | Characterization |
| *OsMADS1* | LOC_Os03g11614 | AK070981 | *OsMADS1* controls differentiation of specific cell types in the lemma and palea and is an early-acting regulator of inner floral organs *OsMADS1* (Prasad et al., 2001). |
| *OsMADS2* | LOC_Os01g66030 | AK070894 | *OsMADS2* unctions control size, shape and differentiation of the highly derived rice floret second-whorl organ (Prasad and Vijayraghavan, 2003). |
| *OsMADS3* | LOC_Os01g10504 | AK108568 | *OsMADS3* plays a synergistic role with *OsMADS13* in both ovule development and floral meristem termination (Li et al*.*, 2011). |
| *OsMADS4* | LOC_Os05g34940 | AK100233 | *OsMADS4* specifies the identity of petals and stamens (Kang and An, 2005). |
| *OsMADS5* | LOC_Os06g06750 | AK064184 | *OsMADS5* exhibits the phenotype of weak dwarfism and early flowering (Kim et al*.*, 2007). |
| *OsMADS6* | LOC_Os02g45770 | AK069103 | The *AGL6*-like gene *OsMADS6* regulates floral organ and meristem identities in rice and controls rice floret fertility (Li et al*.*, 2010; Zhang et al*.*, 2010). |
| *OsMADS7/45* | LOC_Os08g41950 | AK100263 | The *OsMADS7* is homologous to *OM1* (An, 1999). |
| *OsMADS8/24* | LOC_Os09g32948 | AK072867 | The *OsMADS* *8* is homologous to *FBP2* (Kang et al*.*, 1997). |
| *OsMADS13* | LOC_Os12g10540 | AK070425 | *OsMADS13* controls ovule identity in rice (Lopez-Dee et al*.*, 1999). |
| *OsMADS14* | LOC_Os03g54160 | AK121171 | *OsMADS14* is detectable in sterile lemmas, paleas/lemmas, stamens, and carpels (Moon et al*.*, 1999). |
| *OsMADS15* | LOC_Os07g01820 | AK072683 | *OsMADS15* modifies plant architecture and flowering (Lu et al*.*, 2012). |
| *OsMADS16* | LOC_Os06g49840 | AK069317 | Ectopic expression of *OsMADS16* leads to alteration of floral organ identity in rice (Lee et al*.*, 2003a). |
| *OsMADS17* | LOC_Os04g49150 | AK070540 | *OsMADS17* is supposed to be a duplication copy of *OsMADS6* (Zhang et al., 2010). |
| *OsMADS18/28* | LOC_Os07g41370 | AK064704 | Overexpression of *OsMADS18* induces early flowering in rice (Fornara et al*.*, 2004). |
| *OsMADS20* | LOC_Os12g31748 | AY250075 | *OsMADS14*, *OsMADS15*, *OsMADS18* and *OsMADS20* show high sequence homology with *AP1* (Lu et al*.*, 2012). |
| *OsMADS21* | LOC_Os01g66290 | AK070958 | A paralogue of *OsMADS13* (Lee et al*.*, 2003a). |
| *OsMADS22* | LOC_Os02g52340 | AK070121 | *OsMADS22* is expressed in non-vegetative tissues and its ectopic expression induces spikelet meristem indeterminacy (Lee et al*.*, 2012). |
| *OsMADS23* | LOC_Os08g33488 | NA | an *ANR1*-like gene |
| *OsMADS25* | LOC_Os04g23910 | AK102927 | an *ANR1*-like gene |
| *OsMADS26* | LOC_Os08g02070 | AK069122 | *OsMADS26* negatively regulates resistance to pathogens and drought tolerance in rice (Lee et al*.*, 2008). |
| *OsMADS27* | LOC_Os02g36924 | NA | an *ANR1*-like gene |
| *OsMADS29* | LOC_Os02g07430 | AK1095222 | *OsMADS29* controls the degeneration of cells in maternal tissues during seed development of Rice (Yang et al*.*, 2012). |
| *OsMADS30* | LOC_Os06g45650 | NA | *OsMADS29*, *OsMADS30*, and *OsMADS31* are homologous to the *Arabidopsis* *ABS.* |
| *OsMADS31* | LOC_Os04g52410 | NA |  |
| *OsMADS32* | LOC_Os01g52680 | NA | *OsMADS32* plays an important role in regulating rice floral meristem and organs identity (Sang et al*.*, 2012). |
| *OsMADS33* | LOC_Os12g10520 | NA | *OsMADS33* is expressed preferentially in the roots (Lee et al*.*, 2003b). |
| *OsMADS34* | LOC_Os03g54170 | AK100227 | The *SEP*-like gene *OsMADS34* is involved in rice inflorescence and spikelet development (Gao et al*.*, 2010). |
| *OsMADS37* | LOC_Os08g41960 | NA |  |
| *OsMADS47* | LOC_Os03g08754 | NA |  |
| *OsMADS50* | LOC_Os03g03100 | AK104921 | *OsMADS50* controls various floral regulators in rice (Lee et al*.*, 2004). |
| *OsMADS51* | LOC_Os01g69850 | AB003327 | *OsMADS51* is a short-day flowering promoter that functions upstream of *Ehd1*, *OsMADS14*, and *Hd3a* (Kim et al., 2007). |
| *OsMADS55* | LOC_Os06g11330 | AK111859 | *OsMADS55* plays an important role in floral transition and floral meristem identity (Lee et al*.*, 2012). |
| *OsMADS56* | LOC_Os10g39130 | AK070135 | *OsMADS50* and *OsMADS56* function antagonistically in regulating long day (LD)-dependent flowering in rice (RYU et al*.*, 2009). |
| *OsMADS57* | LOC_Os02g49840 | AK108784 | *OsMADS57*, is reported to interact with *OsTB1*, and targets *D14* (*Dwarf 14*) to control the outgrowth of axillary buds (Guo et al*.*, 2013). |
| *OsMADS58* | LOC_Os05g11414 | AK111723 | *OsMADS3* and *OsMADS58* were produced by a recent gene duplication event in plant evolution (Yamaguchi et al*.*, 2006). |
| *OsMADS59* | LOC_Os06g23950 | NA |  |
| *OsMADS60* | LOC_Os02g01360 | AK121824 |  |
| *OsMADS61* | LOC_Os04g38770 | NA | *OsMADS61* promoter was only active in the leaf tips and the stem base (Puig et al*.*, 2013). |
| *OsMADS62* | LOC_Os08g38590 | NA | *OsMADS62*, *OsMADS63* and *OsMADS68* are all specifically expressed late in pollen development (Liu et al*.*, 2013). |
| *OsMADS63* | LOC_Os06g11970 | AK111776 | *OsMADS62*, *OsMADS63* and *OsMADS68* are all specifically expressed late in pollen development (Liu et al*.*, 2013). |
| *OsMADS64* | LOC_Os04g31804 | NA |  |
| *OsMADS65* | LOC_Os01g69850 | AK066160 |  |
| *OsMADS66* | LOC_Os05g11380 | NA |  |
| *OsMADS67* | LOC_Os12g31010 | ABA98556 |  |
| *OsMADS68* | LOC_Os11g43740 | NA | *OsMADS62*, *OsMADS63* and *OsMADS68* are all specifically expressed late in pollen development (Liu et al*.*, 2013). |
| *OsMADS69* | LOC_Os08g20460 | NA |  |
| *OsMADS70* | LOC_Os05g23780 | NA |  |
| *OsMADS71* | LOC_Os06g22760 | NA |  |
| *OsMADS72* | LOC_Os03g14850 | NA | *OsMADS72* participates in flower development (Alvarez-Buylla et al*.*, 2000). |
| *OsMADS73* | LOC_Os12g21850 | NA |  |
| *OsMADS74* | LOC_Os12g21880 | NA |  |
| *OsMADS75* | LOC_Os06g30810 | NA |  |
| *OsMADS76* | LOC_Os06g30830 | NA |  |
| *OsMADS77* | LOC_Os09g02780 | NA |  |
| *OsMADS78* | LOC_Os09g02830 | NA |  |
| *OsMADS79* | LOC_Os01g74440 | NA |  |
| *OsMADS80* | LOC_Os02g06860 | NA |  |
| *OsMADS81* | LOC_Os04g24790 | NA |  |
| *OsMADS82* | LOC_Os04g24800 | NA | . |
| *OsMADS83* | LOC_Os04g24810 | NA |  |
| *OsMADS84* | LOC_Os04g25870 | NA |  |
| *OsMADS85* | LOC_Os04g25920 | NA |  |
| *OsMADS86* | LOC_Os03g37670 | NA |  |
| *OsMADS87* | LOC_Os03g38610 | NA | The gene *OsMADS87* act as a determinant of seed size and thermal sensitivity (Chen et al*.*, 2016). |
| *OsMADS88* | LOC_Os01g18420 | NA |  |
| *OsMADS89* | LOC_Os01g18440 | NA |  |
| *OsMADS90* | LOC_Os07g04170 | NA |  |
| *OsMADS91* | LOC_Os01g11510 | NA |  |
| *OsMADS92* | LOC_Os01g23750 | NA |  |
| *OsMADS93* | LOC_Os01g23760 | NA |  |
| *OsMADS94* | LOC_Os01g23770 | NA |  |
| *OsMADS95* | LOC_Os01g23780 | NA |  |
| *OsMADS96* | LOC_Os01g67890 | NA |  |
| *OsMADS97* | LOC_Os01g68420 | NA |  |
| *OsMADS98* | LOC_Os01g68560 | NA |  |
| *OsMADS99* | LOC_Os04g25930 | NA |  |
| *PISTILLATA* | At5g20240 | NM_122031 | Required for the specification of petal and stamen identities (Goto and Meyerowitz, 1994). |
| *APETALA3* | At3g54340 | NM_115294 | Specifies petal and stamen identities (Jack et al., 1992). |
| *AGAMOUS* | At4g18960 | NM_118013 | Specifies floral meristem and carpel and stamen identity (Sieburth and Meyerowitz, 1997). |
| *AGL1=**SHP1* | At3g58780 | NM_115740 | Two genes (*SHP1* and *SHP2*) are required for fruit dehiscence (Flanagan et al*.*, 1996). |
| *AGL2=**SEP1* | At5g15800 | NM_121585 | *AGL2* is functionally redundant with *SEP2* and *SEP3* (Flanagan and Ma, 1994). |
| *AGL3* | At2g03710 | NM_201682 | *AGL3*, a *SEP* gene, is involved in the development of sepals, petals, stamens and carpels (Huang et al*.*, 1995). |
| *AGL4=**SEP2* | At3g02310 | NM_111098 |  |
| *AGL5=**SHP2* | At2g42830 | NM_180046 | *AGL5*, an *AGAMOUS* -like gene, is involved in fruit development (Kempin et al*.*, 1997). |
| *AGL6* | At2g45650 | NM_130127 | *AGL6* negatively regulates the *FLC*/*MAF* clade genes and positively regulates *FT* in *Arabidopsis*.(Sun et al*.*, 2014) |
| *AGL7=AP1* | At1g69120 | NM_105581 | *AGL7* regulates the expression of flowering time genes *SVP*, *SOC1* and *AGL24* (Mandel et al*.*, 1992). |
| *AGL8=**FUL* | At5g60910 | NM_125484 | The *FRUITFULL* MADS-box gene mediates cell differentiation during *Arabidopsis* fruit development (Gu et al*.*, 1998). |
| *AGL9=**SEP3* | At1g24260 | NM_102272 | *SEP3* act as a downstream gene of miR156-*SPL3*-*FT* circuitry in ambient temperature-responsive flowering (Hwan Lee et al*.*, 2012). |
| *AGL10=CAL* | At1g26310 | NM_102395 | *CAL* enhances the flower to shoot transformation in *ap1* mutants. |
| *AGL11* | At4g09960 | NM_117064 | *AGL11* is expressed in the carpel and ovules and involved in fertilization and seed development. |
| *AGL12* | At1g71692 | NM_105825 | *AGL12* is involved in root cell differentiation and flowering time (Lee et al*.*, 2008). |
| *AGL13* | At3g61120 | NM_115976 | *AGL13* is expressed in both pollen and ovules and involved in male and female gametophyte morphogenesis (Schauer et al*.*, 2007). |
| *AGL14* | At4g11880 | NM_117258 | *AGL14* is an important component of the complex gene regulatory network that underlies *Arabidopsis* shoot apical meristem transitions (Pérez-Ruiz et al*.*, 2015). |
| *AGL15* | At5g13790 | NM_121382 | *AGL15* delayed in the transition to flowering, perianth abscission and senescence and fruit and seed maturation (Heck et al*.*, 1995). |
| *AGL16* | At3g57230 | NM_115583 | *AGL16* controls flowering via a allelic dosage effect in long-day non-vernalized conditions (He et al*.*, 2004). |
| *AGL17* | At2g22630 | NM_127828 | *AGL17* is involved in promoting flowering (Puig et al*.*, 2013). |
| *AGL18* | At3g57390 | NM_115599 | *AGL18,* most similar to *AGL15*, is expressed in endosperm and developing gametophytes (Adamczyk et al*.*, 2007). |
| *AGL19* | At4g22950 | NM_118424 | *AGL19* activates *LFY* and *AP1* and eventually cause flowering (Schönrock et al*.*, 2006). |
| *AGL20=**SOC1* | At2g45660 | NM_130128 | Overexpression of (*SOC1*) *AGL20* suppresses the late flowering of plants and the delayed phase transitions during the vegetative stages of development (Dorca‐Fornell et al*.*, 2011). |
| *AGL21* | At4g37940 | NM_119955 | *AGL21* may be involved in various environmental and physiological signals-mediated lateral root development and growth (Yu et al*.*, 2014). |
| *AGL22=**SVP* | At2g22540 | NM_127820 | *SVP*, as a floral repressor, represses *FT* expression via direct binding to the vCArG III motif in the *FT* promoter (Hartmann et al*.*, 2000). |
| *AGL23* | At1g65360 | [NM_105210](http://www.ncbi.nlm.nih.gov/entrez/viewer.cgi?db=nucleotide&val=NM_105210) | *AGL23* controls female gametophyte development and the biogenesis of organelles during embryo development (Colombo et al*.*, 2008). |
| *AGL24* | At4g24540 | NM_118587 | *AGL24*, *which is* involved in flowering, regulates the expression of *SOC1* and is also upregulated by *SOC1* (Michaels et al., 2003). |
| *AGL25=**FLC* | At5g10140 | NM_121052 | In*Arabidopsis*, *FLOWERING LOCUS C* (*FLC*) is a repressor of flowering (Rouse et al*.*, 2002). |
| *AGL26* | At5g26870 | BT004581 | *AGL26* were identified as root-specific gene (Par̆enicová et al*.*, 2003). |
| *AGL27=**FLM* | At1g77080 | NM_202431 | *FLM* is a negative regulator of flowering (Scortecci et al*.*, 2001). |
| *AGL28* | At1g01530 | NM_105210 | *AGAMOUS*-LIKE *28* (*AGL28*) promotes flowering by upregulating expression of floral promoters within the autonomous pathway (Yoo et al*.*, 2006). |
| *AGL29* | At2g34440 | NM_128996 |  |
| *AGL30* | At2g03060 | NM_126358 | *AGL30* is expressed in pollen and heterodimers with other MICK family members (Adamczyk and Fernandez, 2009). |
| *AGL31* | At5g65050 | NM_125904 | As an *Agamous*-like gene, *AGL31* is involved in control of flowering time (Sreekantan and Thomas, 2006). |
| *AGL32=**TT16* | At5g23260 | NM_122232 | *TT16* regulates proanthocyanidin biosynthesis in the inner-most cell layer of the seed coat (Bogs et al*.*, 2007). |
| *AGL33* | At2g26320 | [NM_128189](http://www.ncbi.nlm.nih.gov/entrez/viewer.cgi?db=nucleotide&val=NM_128189) |  |
| *AGL34* | At5g26575 | CW836839 |  |
| *AGL35* | At5g26625 | BH613122 |  |
| *AGL36* | At5g26645 | AY141247 |  |
| *AGL37* | At1g65330 | NM_105207 |  |
| *AGL38* | At1g65300 | NM_105204 | *AGL38* encodes *PHERES2*, a homolog of *PHERES1*. *PHERES1* and *PHERES2* are both target genes of the *FIS* Polycomb group complex (Villar et al*.*, 2009). |
| *AGL39* | At5g27130 | NM_122595 |  |
| *AGL40* | At4g36590 | NM_119822 |  |
| *AGL41* | At2g26880 | NM_128245 |  |
| *AGL42* | At5g62165 | NM_125610 | As a *SOC1*-like gene, AGL42, promotes flowering in the shoot apical and axillary meristems (Dorca-Fornell et al*.*, 2011). |
| *AGL43* | At5g40220 | NM_123386 |  |
| *AGL44=**ANR1* | At2g14210 | NM_126990 | *ANR1* and other MADS box genes are involved in the nutritional regulation of lateral root growth (Gan et al*.*, 2005). |
| *AGL45* | At3g05860 | NM_202503 |  |
| *AGL46* | At2g28700 | NM_128431 |  |
| *AGL47* | At5g55690 | NM_124951 |  |
| *AGL48* | At2g40210 | NM_129579 |  |
| *AGL49* | At1g60040 | NM_104696 |  |
| *AGL50* | At1g59810 | NM_104674 |  |
| *AGL51* | At4g02240 | AY141215 |  |
| *AGL52* | At4g11250 | NM_117196 |  |
| *AGL53* | At5g27070 | NM_122589 |  |
| *AGL54* | At5g27090 | NM_122591 |  |
| *AGL55* | At1g60920 | NM_104772 |  |
| *AGL56* | At1g60880 | NM_104769 | *AGL56* might play a key role in this N signaling pathway (Gan et al*.*, 2010). |
| *AGL57* | At3g04100 | NM_111281 |  |
| *AGL58* | At1g28450 | NM_102613 |  |
| *AGL59* | At1g28460 | NM_102614 |  |
| *AGL60* | At1g72350 | NM_105894 |  |
| *AGL61* | At2g24840 | NM_179727 | Localized to the nucleus in the central cell and endosperm of the female gametophyte (Kang et al*.*, 2008). |
| *AGL62* | At5g60440 | NM_125437 | *AGL62* supresses suppresses cellularization during the syncytial phase of endosperm development (Kang et al*.*, 2008). |
| *AGL63=GOA* | At1g31140 | NM_102852 | *GOA* (*AGL63*) represses fruit growth and contributes to integument development (Erdmann et al*.*, 2010). |
| *AGL64* | At1g29960 | NM_102736 |  |
| *AGL65* | At1g18750 | NM_101733 | *AGL65* is involved in late stages of pollen development and pollen tube growth (Adamczyk and Fernandez, 2009). |
| *AGL66* | At1g77980 | NM_106447 | *AGL66* is involved in late stages of pollen development and pollen tube growth (Adamczyk and Fernandez, 2009). |
| *AGL67* | At1g77950 | NM_106444 |  |
| *AGL68* | At5g65080 | NM_125907 | *AGL68* is upregulated during vernalization and regulates flowering time (Posé et al*.*, 2013). |
| *AGL69* | At5g65070 | NM_125906 | *AGL69* regulates flowering time (Posé et al*.*, 2013). |
| *AGL70* | At5g65060 | NM_125905 | *AGL70* is closely related to *FLC* (Agarwal et al., 2011)*.* |
| *AGL71* | At5g51870 | NM_203195 | *AGL71* promotes flowering in the shoot apical and axillary meristems (Dorca-Fornell et al*.*, 2011). |
| *AGL72* | At5g51860 | NM_124565 | As a *SOC1*-like genes, AGL72, promotes flowering in the shoot api Axillary meristems (Dorca-Fornell et al*.*, 2011). |
| *AGL73* | At5g38620 | NM_123223 |  |
| *AGL74* | At1g48150 | NM_103711 |  |
| *AGL75* | At5g41200 | NM_123485 |  |
| *AGL76* | At5g40120 | NM_123375 |  |
| *AGL77* | At5g38740 | NM_123235 |  |
| *AGL78* | At5g65330 | NM_125931 |  |
| *AGL79* | At3g30260/At3g30270 | NM_113925 |  |
| *AGL80* | At5g48670 | NM_124244 | *AGL80* controls the expression of downstream genes required for central cell development and function (Portereiko et al*.*, 2006). |
| *AGL81* | At5g39750 | NM_123337 |  |
| *AGL82* | At5g58890 | NM_125279 |  |
| *AGL83* | At5g49490 | NM_124326 |  |
| *AGL84* | At5g49420 | NM_124319 |  |
| *AGL85* | At1g54760 | NM_104351 |  |
| *AGL86* | At1g31630 | NM_102898 |  |
| *AGL87* | At1g22590 | NM_202157 |  |
| *AGL88* | At2g11990 | AY233210 |  |
| *AGL89* | At5g27580 | NM_122640 |  |
| *AGL90* | At5g27960 | NM_122679 |  |
| *AGL91* | At3g66656 | NM_111544 |  |
| *AGL92* | At1g31640 | NM_102899 | A paternally expressed imprinted gene (Hsieh et al*.*, 2011). |
| *AGL93* | At5g26950 | NM_122577 |  |
| *AGL94* | At1g69540 | NM_105623 |  |
| *AGL95* | At2g15660 | NM_127127 |  |
| *AGL96* | At5g06500 | NM_120733 |  |
| *AGL97* | At1g46408 | NM_103604 |  |
| *AGL98* | At5g39810 | NM_123344 |  |
| *AGL99* | At5g04640 | NM_120546 |  |
| *AGL100* | At1g17310 | NM_101593 |  |
| *AGL101* | At5g27050 | NM_122587 |  |
| *AGL102* | At1g47760 | NM_103669 |  |
| *AGL103* | At3g18650 | NM_112751 |  |
| *AGL104* | At1g22130 | NM_102063 | *AGL104* is expressed in pollen and then involved in late stages of pollen development and pollen tube growth (Adamczyk and Fernandez, 2009). |
| *AGL105* | At5g37420 | AY141227 |  |

**Table S2** The accession numbers of rice MADS-box genes are from Rice Genome Annotation Project (http://rice.plantbiology.msu.edu/index.html), of *Arabidopsis* MADS-box genes are from TIAR (The *Arabidopsis* Information Resource; http://www.arabidopsis.org/).

| Table S3 Sequence and length of motifs identified from *P. edulis* MADS-box proteins | | |
| --- | --- | --- |
| Motif | Motif Length (aa) | Sequence |
| 1 | 57 | GRGKVEIKRIENPTNRQVTFSKRRNGLFKKAYELSVLCDAEVALIVFSPTGKLYEYA |
| 2 | 35 | HLMGEDLNSCSPEELHQLEQQLENGLHNIRSRKTQ |
| 3 | 57 | MLEDEHKLLAFRMHQQDVELSGGMRELELGYHHGRDFAPQMPFTFRVQPSHPNLQED |
| 4 | 15 | SMDKIIDRYWTYSYD |
| 5 | 41 | NQLPLVTPAPMTMAPPDENPDPNNDHMDVETELYIGLPGRD |
| 6 | 41 | MELQNDNMYLRSKVAENERGQQPMNMMGAASTSEYDHMVPY |
| 7 | 21 | GNWCDEYGKLKHKIETIQKCQ |
| 8 | 29 | IEWDKKHKSLSEENDRIQKENDNMQIELR |
| 9 | 29 | NKTVQQDIEQVKADAVGLAKKLEALEDYK |
| 10 | 21 | KLKEKEMTLLKDNEDLREKCK |
| 11 | 38 | NVITEDGQSSESVMTALHPGSPQDNDDGSDISLKLGLP |
| 12 | 28 | DSRNFLQVNIMQQQPQHYSHQLQPTALQ |
| 13 | 8 | HWRMHRRN |
| 14 | 18 | TNDLNIEARDSRVDCNIQ |
| 15 | 10 | PCGPFCHCFP |
| 16 | 7 | WPQTWPQ |
| 17 | 9 | PPWMVRCIN |
| 18 | 36 | FSGPYDSCGPGDEANQPNWHSAVCDPSLHPCYQQPY |
| 19 | 9 | YAEVEYMQK |
| 20 | 6 | WWFHPG |

**Table S3** Sequence and length of motifs identified from *P. edulis* MADS-box proteins using MEME motif search tool (aa, amino acids).

| Table S4 DGE analysis of *P. edulis* MADS genes during floral organ development | | | | | | |
| --- | --- | --- | --- | --- | --- | --- |
| ID | Gene name | CK1_RPKM | F1_RPKM | F2_RPKM | F3_RPKM | F4_RPKM |
| PH01005177G0100 | *PheMADS26* | 0.01 | 2.198097 | 2.123223 | 5.25528 | 2.699941 |
| PH01000338G0120 | *PheMADS31* | 0.01 | 0.879517 | 1.699115 | 7.359717 | 2.520741 |
| PH01000794G0210 | *PheMADS4-1* | 0.083828 | 6.795582 | 17.58242 | 27.41697 | 2.086767 |
| PH01002127G0260 | *PheMADS3* | 0.430171 | 22.66684 | 36.57144 | 145.1215 | 24.09393 |
| PH01000006G0210 | *PheMADS20* | 0.040721 | 2.122108 | 0.683274 | 0.01 | 0.01 |
| PH01000606G0250 | *PheMADS15* | 3.757258 | 131.1953 | 136.597 | 227.9102 | 158.3646 |
| PH01000222G1190 | *PheMADS14* | 2.071772 | 62.01205 | 59.36492 | 49.99927 | 40.26691 |
| PH01001952G0230 | *PheMADS5* | 1.73811 | 42.77346 | 128 | 122.8172 | 155.0415 |
| PH01001878G0200 | *PheMADS4-2* | 1.478225 | 35.18908 | 46.85169 | 72.95158 | 45.31605 |
| PH01001174G0480 | *PheMADS90* | 0.316093 | 6.589086 | 8.132599 | 21.44212 | 6.68832 |
| PH01000222G1060 | *PheMADS34* | 0.643644 | 10.76675 | 22.00001 | 11.2619 | 5.785889 |
| PH01000306G0610 | *PheMADS18-1* | 12.37755 | 159.8416 | 209.681 | 157.4846 | 109.4487 |
| PH01001278G0330 | *PheMADS21* | 0.827759 | 8.740275 | 14.70639 | 76.38186 | 21.21177 |
| PH01000117G1210 | *PheMADS37-1* | 3.763999 | 35.30796 | 32.42106 | 11.85463 | 24.83013 |
| PH01001750G0200 | *PheMADS91* | 0.459373 | 3.869017 | 3.270074 | 12.39378 | 3.898409 |
| PH01000216G0990 | *PheMADS1* | 3.632558 | 26.29243 | 34.53969 | 51.70299 | 37.30097 |
| PH01002743G0050 | *PheMADS18-2* | 47.80036 | 195.5648 | 250.3226 | 144.197 | 121.7477 |
| PH01000466G0340 | *PheMADS68* | 0.123703 | 0.358146 | 2.421622 | 20.41658 | 0.192462 |
| PH01000317G0080 | *PheMADS2* | 7.774369 | 20.2892 | 42.5646 | 138.2764 | 3.747944 |
| PH01000038G1550 | *PheMADS47* | 3.034765 | 5.473398 | 6.400002 | 5.423792 | 12.38451 |
| PH01003178G0150 | *PheMADS65* | 33.79546 | 60.09349 | 67.72095 | 53.18679 | 60.44649 |
| PH01000437G0930 | *PheMADS22* | 27.50229 | 40.68407 | 28.07018 | 45.29077 | 23.11232 |
| PH01000616G0020 | *PheMADS33* | 0.963584 | 1.394883 | 1.684211 | 0.729516 | 0.01 |
| PH01001303G0110 | *PheMADS37-2* | 47.07052 | 47.6849 | 53.33335 | 18.90109 | 15.64483 |
| PH01000954G0350 | *PheMADS72* | 0.091055 | 0.01 | 0.169761 | 0.01 | 1.322219 |
| PH01003236G0170 | *PheMADS29* | 0.063925 | 0.01 | 4.648046 | 10.0665 | 13.92393 |
| PH01000059G1270 | *PheMADS56-3* | 214.9861 | 67.45074 | 46.70271 | 40.58343 | 31.43546 |
| PH01002152G0120 | *PheMADS56-4* | 135.1581 | 50.5127 | 44.03962 | 33.62273 | 21.50429 |
| PH01000107G0570 | *PheMADS56-2* | 179.3408 | 75.26272 | 70.62785 | 28.93274 | 25.58063 |
| PH01000759G0450 | *PheMADS56-1* | 167.5373 | 74.46574 | 66.2655 | 32.19876 | 22.37158 |
| PH01002755G0230 | *PheMADS50-2* | 42.28297 | 20.8236 | 15.2381 | 17.82103 | 10.17299 |
| PH01002152G0090 | *PheMADS50-1* | 70.68163 | 40.7204 | 30.00001 | 64.25059 | 59.34245 |
| PH01000080G0200 | *PheMADS55* | 22.75258 | 15.34909 | 6.424712 | 2.408247 | 7.423534 |
| PH01000518G0720 | *PheMADS64* | 2.22907 | 1.720959 | 1.662338 | 0.01 | 1.849635 |

**Table S4** DGE analysis of *P. edulis* MADS genes during floral organ development. The common names and gene IDs of PheMADS genes from different visions during floral organ development. The RPKM values of CK1, F1, F2, F3 and F4 were listed.

| Table S5 Primer sets used for the qPCRs | | |
| --- | --- | --- |
| Gene name | Forward primer (5'---3') | Reverse primer (5'---3') |
| *PheMADS1* | CGTACGAGTTGTCGGTGCTCT | CTACCATGAGGATGTGGAGAACTCG |
| *PheMADS3* | AAGATGGGGAGGGGCAAGATAGAG | AGCACCGAGAGTTCATACGCCTTC |
| *PheMADS15* | TGCTCAAGAAGGCGCATGAGA | GTCGGTGGCGTACTCGCAG |
| *PheMADS20* | GGAGTTGTGTGTTGTTGCGCTC | GCTCTCCCATAAGATTCCTTTGGCT |
| *PheMADS22* | TGGCAAGCAAGTGGTTGCTGATA | GTCAGGGATACATCCGAACCATCA |
| *PheMADS50-1* | CGCGGCAAGCTCTAC GAG TTC | GAGGAGGATGGGCAGTTGGTAC |
| *PheMADS56-1* | TCGCGGCGCAAGATGAGAAC | TAGCCCGATTATCTGTTGTCGCC |
| *PheMADS90* | TGTGCTGCATATGTCTTGCCGTG | AGGGAAGGATCACAAACTGCAGAG |
| *LFY* | GGTGCAGCAACAAGACCAGA | CCGTCGTCATCCTCACCTTC |
| *TFL1* | TGGCCATGAGCTCTTTCCTT | ATCTGTTGTGCCGGGAATGT |
| *TIP41* | AAAATCATTGTAGGCCATTGTCG | ACTAAATTAAGCCAGCGGGAGTG |

**Table S5** Primers for quantitative real-time RT-PCR (qRT-PCR) in this study. F and R represented the forward and reverse primers, respectively. qPCR of flowering tissues at different flowering developmental stages and leaves of the seedlings in different stresses, was performed separately using leaves of non-flowering plants and the seedlings without stress as the control, respectively. Tonoplast intrinsic protein (TIP41) (Fan et al*.*, 2013a) and *β*-tubulin were used as controls. The qPCR reactions were carried out with LightCycler480® System (Roche, USA) using SYBR® Premix EX TaqTM kit (Roche, USA). The following amplification reactions were performed for qPCR: 95°C for 10 s, 60°C for 10 s, and 72°C for 20 s. The experiments were repeated three times with independent cDNA samples. Data was analyzed using Roche manager software.

| Table S6 The specific probes and sense controls of *PheMADS15*, *PheMADS4-1*, *PheMADS3*, *PheMADS21* and *PheMADS5* | | |
| --- | --- | --- |
| Gene name | specific probes (5’-3’) | sense controls (5’-3’) |
| *PheMADS15* | GGCAAGCTCTGCGAGTACGCCACCGACTCATGGTGTATTTTCCTCC | GGAGGAAAATACACCATGAGTCGGTGGCGTACTCGCAGAG CTTGCC |
| *PheMADS5* | GGTTTCCCCTCTTTGCTTTCGCCTCGATTAATTGGAGCCGGCCGG | CCGGCCGGCTCCAATTAATCGAGGCGAAAG CAAAGAGGGG AAACC |
| *PheMADS3* | CCATGAGCCTCAGGGATCTTAAGCAGCTAGAGGGCAGGCTGGAG | CTCCAGCCTGCCCTCTAGCTGCTTAAGATC CCTGAGGCTC ATGG |
| *PheMADS4-1* | CCGGGAAGATACTGTGGGATGAGAAGCACAAGAGCCTCAGCGCGG | CCGCGCTGAGGCTCTTGTGCTTCTCATCCC ACAGTATCTT CCCGG |
| *PheMADS21* | CGGCGACAGCGGAGGGTGTGGATAGAGGAGCTAAAGGGAGGAGG | CCTCCTCCCTTTAGCTCCTCTATCCACACCCTCCGCTGTC GCCG |

**Table S6** The specific probes and sense controls of *PheMADS15*, *PheMADS4-1*, *PheMADS3*, *PheMADS21* and *PheMADS5.* These probes were designed and synthesized by GENEWIZ.

**References**

Adamczyk, B.J., and Fernandez, D.E. (2009). MIKC* MADS domain heterodimers are required for pollen maturation and tube growth in *Arabidopsis*. *Plant Physiol*. 149, 1713-1723.

Adamczyk, B.J., Lehti-Shiu M.D., and Fernandez, D.E. (2007). The MADS domain factors AGL15 and AGL18 act redundantly as repressors of the floral transition in *Arabidopsis*. *Plant J*. 50, 1007-1019.

Agarwal, P., Kapoor, S., and Tyagi, A.K. (2011). Transcription factors regulating the progression of monocot and dicot seed development. *Bioessays*, 33, 189-202.

Alvarez-Buylla, E.R., Pelaz, S., Liljegren, S.J., Gold, S.E., Burgeff, C., Ditta, G.S., De Pouplana, L.R., et al. (2000). An ancestral MADS-box gene duplication occurred before the divergence of plants and animals. *Proc. Nat. Acad. Sci. USA*, 97, 5328-5333.

An G. Gene controlling floral development and apical dominance in plants: U.S. *Patent*, 5,859,326.

Bogs, J., Jaffé, F.W., Takos, A.M., Walker, A.R., and Robinson, S.P. (2007). The grapevine transcription factor VvMYBPA1 regulates proanthocyanidin synthesis during fruit development. *Plant Physiol.* 143, 1347-1361.

Chen, C., Begcy, K., Liu, K., Folsom, J.J., Wang, Z., Zhang, C., Walia, H., et al. (2016). Molecular characterization of rice endosperm development under heat stress identifies *OsMADS87* as a determinant of seed size and thermal sensitivity. *Plant Physiol*. 01992.

Colombo, M., Masiero, S., Vanzulli, S., Lardelli, P., Kater, M.M., and Colombo, L. (2008). *AGL23*, a type I MADS-box gene that controls female gametophyte and embryo development in *Arabidopsis*. *Plant J*. 54, 1037-1048.

Dorca-Fornell, C., Gregis, V., Grandi, V., Coupland, G., Colombo, L., and Kater, M.M. (2011). The *Arabidopsis* *SOC1*-like genes *AGL42*, *AGL71* and *AGL72* promote flowering in the shoot apical and axillary meristems. *Plant J.* 67, 1006-1017.

Erdmann, R., Gramzow, L., Melzer, R., Theißen, G., and Becker, A. (2010). *GORDITA* (*AGL63*) is a young paralog of the *Arabidopsis thaliana* Bsister MADS box gene *ABS* (*TT16*) that has undergone neofunctionalization. *Plant J*. 63, 914-924.

Flanagan, C.A., Hu, Y., and Ma, H. (1996). Specific expression of the *AGL1* MADS-box gene suggests regulatory functions in *Arabidopsis* gynoecium and ovule development. *Plant J*. 10, 343-353.

Flanagan, C.A., and Ma, H. (1994). Spatially and temporally regulated expression of the MADS-box gene *AGL2* in wild-type and mutant *Arabidopsis* flowers. *Plant Mol. Bio*. 26, 581-595.

Fornara, F., Pařenicová, L., Falasca, G., Pelucchi, N., Masiero, S., Ciannamea, S., Lopez-Dee, Z., et al. (2004). Functional characterization of *OsMADS18*, a member of the *AP1*/*SQUA* subfamily of MADS box genes. *Plant Physiol*. 135, 2207-2219.

Gan, Y., Filleur, S., Rahman, A., Gotensparre, S., and Forde, B.G. (2005). Nutritional regulation of *ANR1* and other root-expressed MADS-box genes in *Arabidopsis thaliana*. *Planta*, 222, 730-742.

Gan, Y., Zhou, Z., An, L., Bao, S., Liu, Q., Srinivasan, M., and Goddard, P. (2010). The effects of fluctuations in the nutrient supply on the expression of *ANR1* and 11 other MADS box genes in shoots and roots of *Arabidopsis thaliana*. *Botany*, 88, 1023-1031.

Gao, X., Liang, W., Yin, C., Ji, S., Wang, H., Su, X., Guo, C., Kong, H., et al. (2010). The *SEPALLATA-like* gene *OsMADS34* is required for rice inflorescence and spikelet development. *Plant Physiol*. 153, 728-740.

Goto, K., and Meyerowitz, E.M. (1994). Function and regulation of the *Arabidopsis* floral homeotic gene *PISTILLATA*. *Gene. Dev*. 8, 1548-1560.

Gu, Q., Ferrándiz, C., Yanofsky, M.F., and Martienssen, R. (1998). The *FRUITFULL* MADS-box gene mediates cell differentiation during *Arabidopsis* fruit development. *Development*, 125, 1509-1517.

Guo, S., Xu, Y., Liu, H., Mao, Z., Zhang, C., Ma, Y., Zhang, Q., et al. (2013). The interaction between *OsMADS57* and *OsTB1* modulates rice tillering via *DWARF14*. *Nat. Commun*. 4, 1566.

Hartmann, U., Höhmann, S., Nettesheim, K., Wisman, E., Saedler, H., and Huijser, P. (2000). Molecular cloning of *SVP*: a negative regulator of the floral transition in *Arabidopsis*. *Plant J*. 21, 351-360.

He, Y., Doyle, M.R., and Amasino, R.M. (2004). PAF1-complex-mediated histone methylation of *FLOWERING LOCUS C* chromatin is required for the vernalization-responsive, winter-annual habit in *Arabidopsis*. *Gene. Dev*. 18, 2774-2784.

Heck, G.R., Perry, S.E., Nichols, K.W., and Fernandez, D.E. (1995). *AGL15*, a MADS domain protein expressed in developing embryos. *Plant Cell*, 7, 1271-1282.

Hsieh, T.F., Shin, J., Uzawa, R., Silva, P., Cohen, S., Bauer, M.J., Hashimoto, M., et al. (2011). Regulation of imprinted gene expression in *Arabidopsis* endosperm. *Proc. Nat. Acad. Sci. USA*. 108, 1755-1762.

Huang, H., Tudor, M., Weiss, C.A., Hu ,Y., and Ma, H. (1995). The *Arabidopsis* MADS-box gene *AGL3* is widely expressed and encodes a sequence-specific DNA-binding protein. *Plant Mol. Bio*. 28, 549-567.

Hwan, L.J., Joon K.J., and Ahn, J.H. (2012). Role of *SEPALLATA3* (*SEP3*) as a downstream gene of miR156-SPL3-FT circuitry in ambient temperature-responsive flowering. *Plant Signal Behav*. 7, 1151-1154.

Jack, T., Brockman, L.L., and Meyerowitz, E.M. (1992). The homeotic gene *APETALA3* of *Arabidopsis thaliana* encodes a MADS box and is expressed in petals and stamens. *Cell*, 68, 683-697.

Kang, H.G., and An, G. (2005). Morphological alterations by ectopic expression of the rice *OsMADS4* gene in tobacco plants. *Plant Cell Rep*. 24, 120-126.

Kang, H., Jang, S., Chung, J., Cho, Y., and An, G. (1997). Characterization of two rice MADS box genes that control flowering time. *Mol. Cells*, 7, 559-566.

Kang, I.H., Steffen, J.G., Portereiko, M.F., Lloyd, A., and Drews, G.N. (2008). The *AGL62* MADS domain protein regulates cellularization during endosperm development in *Arabidopsis*. *Plant Cell*, 20, 635-647.

Kater, M.M., Dreni, L., and Colombo, L. (2006). Functional conservation of MADS-box factors controlling floral organ identity in rice and *Arabidopsis*. *J**. Exp. Bot*. 57, 3433-3444.

Kempin, S., Liljegren, S., Block, L., Rounsley, S., Lam, E., and Yanofsky, M. (1997). Inactivation of the *Arabidopsis AGL5* MADS-box gene by homologous recombination. *Nature*, 389, 802-803.

Kim, S.L., Lee, S., Kim, H.J., Nam, H.G., and An, G. (2007). *OsMADS51* is a short-day flowering promoter that functions upstream of *Ehd1*, *OsMADS14* and *Hd3a*. *Plant Physiol.* 145, 1484-1494.

Lee, J.H., Park, S.H., and Ahn, J.H. (2012). Functional conservation and diversification between rice OsMADS22/OsMADS55 and *Arabidopsis* SVP proteins. *Plant Sci*. 185, 97-104.

Lee, S., Jeon, J.S., An, K., Moon, Y.H., Lee, S., Chung, Y.Y., An, G., et al. (2003a). Alteration of floral organ identity in rice through ectopic expression of *OsMADS16*. *Planta*, 217, 904-911.

Lee, S., Kim, J., Han, J.J., Han, M.J., and An, G. (2004). Functional analyses of the flowering time gene *OsMADS50*, the putative *SUPPRESSOR OF OVEREXPRESSION OF CO 1/AGAMOUS-LIKE 20* (*SOC1*/*AGL20*) ortholog in rice. *Plant J*. 38, 754-764.

Lee, S., Kim, J., Son, J.S., Nam, J., Jeong, D.H., Lee, K, Jang, S., et al*.* (2003b). Systematic reverse genetic screening of T-DNA tagged genes in rice for functional genomic analyses: MADS-box genes as a test case. *Plant Cell Physiol*. 44, 1403-1411.

Lee, S., Woo, Y.M., Ryu, S.I., Shin, Y.D., Kim, W.T., Park, K.Y., Lee, I.J., et al*.* (2008). Further characterization of a rice *AGL12* group MADS-box gene, *OsMADS26*. *Plant Physiol*. 147, 156-168.

Li, H., Liang, W., Jia, R., Yin, C., Zong, J., Kong, H., Zhang, D., et al*.* (2010) The *AGL6*-like gene *OsMADS6* regulates floral organ and meristem identities in rice. *Cell Res*. 20, 299-313.

Li, H., Liang, W., Yin, C., Zhu, L., and Zhang, D. (2011). Genetic interaction of *OsMADS3*, *DROOPING LEAF*, and *OsMADS13* in specifying rice floral organ identities and meristem determinacy. *Plant Physiol*. 156, 263-274.

Liu, Y., Cui, S., Wu, F., Yan, S., Lin, X., Du, X., Chong, K., Schilling, S., et al. (2013). Functional conservation of MIKC*-Type MADS box genes in *Arabidopsis* and rice pollen maturation. *Plant Cell*. 25, 1288-1303.

Lopez-Dee, Z.P., Wittich, P., Pe, M.E., Rigola, D., Del, Buono I., Gorla, M.S., Kater, M.M., et al. (1999). *OsMADS13*, a novel rice MADS-box gene expressed during ovule development. *Dev. Gene.* 25, 237-244.

Lu, S.J., Wei, H., Wang, Y., Wang, H.M., Yang, R.F., Zhang, X.B., Tu, J.M., et al. (2012). Overexpression of a transcription factor *OsMADS15* modifies plant architecture and flowering time in rice (*Oryza sativa* L.). *Plant Mol. Bio. Rep*. 30, 1461-1469.

Mandel, M.A., Gustafson-Brown, C., Savidge, B., and Yanofsky, M.F., et al. (1992). Molecular characterization of the *Arabidopsis* floral homeotic gene *APETALA1*. *Nature*, 360, 273-277.

Michaels, S.D., Ditta, G., Gustafson-Brown, C., Pelaz, S., Yanofsky, M., and Amasino, R.M. (2003). *AGL24* acts as a promoter of flowering in *Arabidopsis* and is positively regulated by vernalization. *Plant J*. 33, 867-874.

Moon, Y.H., Jung, J.Y., Kang, H.G., and An G. (1999). Identification of a rice *APETALA3* homologue by yeast two-hybrid screening. *Plant Mol. Bio*. 40, 167-177.

Pérez-Ruiz, R.V., García-Ponce, B., Marsch-Martínez, N., Ugartechea-Chirino, Y., Villajuana-Bonequi, M., de Folter, S., Azpeitia, E., et al*.* (2015). *XAANTAL2* (*AGL14*) is an important component of the complex gene regulatory network that underlies *Arabidopsis* shoot apical meristem transitions. *Mol. Plant*, 8, 796-813.

Par̆enicová, L., de Folter, S., Kieffer, M., Horner, D.S., Favalli, C., Busscher, J., Cook, H.E., et al*.* (2003). Molecular and phylogenetic analyses of the complete MADS-box transcription factor family in *Arabidopsis* new openings to the MADS world. *Plant Cell*. 15, 1538-1551.

Portereiko, M.F., Lloyd, A., Steffen, J.G., Punwani, J.A., Otsuga, D., and Drews, G.N. (2006). *AGL80* is required for central cell and endosperm development in *Arabidopsis*. *Plant Cell*, *18*, *1862-1872.*

Posé, D., Verhage, L., Ott, F., Yant, L., Mathieu, J., Angenent, G.C., Immink, R.G., et al*.* (2013). Temperature-dependent regulation of flowering by antagonistic *FLM* variants. *Nature*, 503, 414-417.

Prasad, K., Sriram, P., Kumar, S.C., Kushalappa, K., and Vijayraghavan, U. (2001). Ectopic expression of rice *OsMADS1* reveals a role in specifying the lemma and palea, grass floral organs analogous to sepals. *Dev. Genes Evol*. 211, 281-290.

Prasad, K., and Vijayraghavan, U. (2003). Double-stranded RNA interference of a rice *PI*/*GLO* paralog, *OsMADS2*, uncovers its second-whorl-specific function in floral organ patterning. *Genetics*, 165, 2301-2305.

Puig, J., Meynard, D., Khong, G.N., Pauluzzi, G., Guiderdoni, E., and Gantet, P. (2013). Analysis of the expression of the AGL17-like clade of MADS-box transcription factors in rice. *Gene Expr. Patterns*, 13, 160-170.

Rouse, D.T., Sheldon, C.C., Bagnall, D.J., Peacock, W.J., and Dennis, E.S. (2002). FLC, a repressor of flowering, is regulated by genes in different inductive pathways. *Plant J*. 29, 183-191.

Ryu, C.H., Lee, S., Cho, L.H., Kim, S.L., Lee, Y.S., Choi, S.C., Jeong, H.J., et al. (2009). *OsMADS50* and *OsMADS56* function antagonistically in regulating long day (LD)-dependent flowering in rice. *Plant Cell Environ*. 32, 1412-1427.

Sang, X., Li, Y., Luo, Z., Ren, D., Fang, L., Wang, N., Zhao, F., et al. (2012). *CHIMERIC FLORAL ORGANS1*, encoding a monocot-specific MADS box protein, regulates floral organ identity in rice. *Plant Physiol*. 160, 788-807.

Schönrock, N, Bouveret, R, Leroy, O, Borghi, L, Köhler, C, Gruissem, W, Hennig, L., et al (2006). Polycomb-group proteins repressthe floral activator *AGL19* in the *FLC-*independent vernalization pathway. *Gene. Dev*. 20, 1667-1678.

Schauer, S.E., Baskar, R., Brand, L., Bolaños, A., Grobei, M., Federer, M., Gagliardini, V., et al*.* (2007). Examination of the role of the *Arabidopsis* MADS-box transcription factors *AGL6* and *AGL13* in reproduction. *Dev. Biol.* 306, 312.

Scortecci, K.C., Michaels, S.D., and Amasino, R.M. (2001). Identification of a MADS-box gene, *FLOWERING LOCUS M*, that represses flowering. *Plant J*. 26, 229-236.

Sieburth, L.E., and Meyerowitz, E.M. (1997). Molecular dissection of the *AGAMOUS* control region shows that cis elements for spatial regulation are located intragenically. *Plant Cell*, 9, 355-365.

Sreekantan, L., and Thomas, M.R. (2006). *VvFT* and *VvMADS8*, the grapevine homologues of the floral integrators *FT* and *SOC1*, have unique expression patterns in grapevine and hasten flowering in *Arabidopsis*. *Funct. Plant Biol*. 33, 1129-1139.

Sun, W., Huang, W., Li, Z., Song, C., Liu, D., Liu, Y., Hayward, A., et al*.* (2014). Functional and evolutionary analysis of the *AP1*/*SEP*/*AGL6* superclade of MADS-box genes in the basal eudicot *Epimedium sagittatum*. *Ann Bot-London* , mct301.

Villar, CBR., Erilova, A., Makarevich, G., Trösch, R., and Köhler, C. (2009). Control of *PHERES1* imprinting in *Arabidopsis* by direct tandem repeats. *Mol. Plant*, 2, 654-660.

Yamaguchi, T., Lee, D.Y., Miyao, A., Hirochika, H., An, G., and Hirano, H.Y. (2006). Functional diversification of the two C-class MADS box genes *OsMADS3* and *OsMADS58* in *Oryza sativa*. *Plant Cell*, 18, 15-28.

Yang, X., Wu, F., Lin, X., Du, X., Chong, K., Gramzow, L., Schilling, S., et al. (2012). Live and let die-the B sister MADS-box gene *OsMADS29* controls the degeneration of cells in maternal tissues during seed development of rice (*Oryza sativa*). *PLoS One*, 7, e51435.

Yoo, S.K., Lee, J.S., and Ahn, J.H. (2006). Overexpression of *AGAMOUS-LIKE 28* (*AGL28*) promotes flowering by upregulating expression of floral promoters within the autonomous pathway. *Biochem. Bioph. Res. Co.* 348, 929-936.

Yu, L.H., Miao, Z.Q., Qi, G.F., Wu, J., Cai, X.T., Mao, J.L., et al. (2014). MADS-Box transcription factor *AGL21* regulates lateral root development and responds to multiple external and physiological signals. *Mol. Plant*, 7, 1653-1669.

Zhang, J., Nallamilli, B.R., Mujahid, H., and Peng, Z. (2010). *OsMADS6* plays an essential role in endosperm nutrient accumulation and is subject to epigenetic regulation in rice (*Oryza sativa*). *Plant J*. 64, 604-617.
